# Multifunctional Agarose-Based Biomaterials: From Tissue Engineering and Immunomodulation to Advanced Diagnostics and Translational Applications

**DOI:** 10.3390/gels12090832

**Published:** 2026-09-11

**Authors:** Zhenzhen Liu, Long Zhang, Jiayuan Xie, Jingyi Zhou, Yang Yang, Ling Wang

**Affiliations:** School of Chemistry and Chemical Engineering, Qilu University of Technology (Shandong Academy of Sciences), Jinan 250353, China; 17657727767@163.com (Z.L.); 19015310881@163.com (L.Z.); xie908557198@163.com (J.X.); z1227318380@163.com (J.Z.); 13678863240@126.com (Y.Y.)

**Keywords:** agarose biomaterials, tissue engineering, drug delivery, immunomodulation, diagnostics, cryopreservation, translational applications

## Abstract

Agarose, a naturally derived marine polysaccharide extracted from red algae, has evolved from a conventional electrophoretic matrix into a multifunctional biomaterial platform for biomedical engineering. Its thermoreversible gelation, tunable pore structure, optical transparency, generally low immunogenicity under tested conditions, and chemical modifiability enable applications in tissue engineering, drug delivery, molecular diagnostics, immunomodulation, and cell preservation. This review critically examines recent advances in agarose-based biomaterials, with emphasis on structure–property relationships, stimulus-responsive delivery systems, regenerative scaffolds, immune–material interactions, agarose-enabled diagnostic microdevices, and DMSO-free cryopreservation. Representative developments include proof-of-concept microfluidic detection of a cfDNA surrogate and histones in spiked plasma, agarose composite hydrogels for controlled release and osteochondral repair, agarose-containing composite hydrogels investigated for macrophage modulation, and agarose/trehalose systems that provide immediate post-thaw viability comparable to conventional DMSO-based preservation in the reported cell model, although post-thaw proliferation remained lower. Agarose is commercially established in electrophoresis and bioseparation, whereas therapeutic delivery and implantable regenerative systems remain predominantly preclinical. Remaining barriers include limited in vivo degradability, insufficient intrinsic cell adhesiveness and bioactivity, trade-offs among mechanical strength, injectability and printability, and incomplete manufacturing and regulatory standardization. Future work should prioritize well-defined degradation pathways, reproducible composition–property relationships, application-specific benchmarking, and clinically relevant validation.

## 1. Introduction

Tissue engineering integrates cells, biomaterial scaffolds, and instructive microenvironments, with formats ranging from thin films to three-dimensional constructs [1,2]. Natural and synthetic polymers provide tunable platforms for this purpose [3]. Among them, carbohydrate-derived polymers are valued for their structural diversity, generally favorable biocompatibility, and potential for chemical functionalization [4,5,6]. These features have stimulated their use in tissue engineering, drug delivery, diagnostics, and other healthcare technologies.

Among carbohydrate polymers, alginate and chitosan are widely investigated; agarose is another important marine polysaccharide platform with distinctive thermoreversible gelation. Agarose can be fabricated as bulk or injectable hydrogels [7,8,9,10,11,12,13,14], self-healing adhesive systems [15], fibers [16,17], and 3D-printed scaffolds [18,19]. It has been investigated for controlled delivery [20,21,22], cartilage and bone repair [14,23,24,25], wound repair [15,26], diagnostic platforms [27,28], and cryopreservation [29].

Agarose has considerable potential in molecular diagnostics and nucleic-acid analysis because its pore structure, optical properties, and thermoreversible processing can be tuned for separation and sensing [27,28]. Recent work includes integrated amplification and point-of-care devices [27,28] as well as RNA enrichment and detection strategies [30].

Recent reviews have discussed agarose-based biomaterials from complementary perspectives. Zarrintaj et al. summarized tissue-engineering applications [31]; Jiang et al. reviewed agarose degradation and valorization pathways [32]; Najihah et al. surveyed reinforced polysaccharide nanocomposite hydrogels [33]; and Huang et al. focused on agarose hydrogels for bone tissue engineering and bioprinting [34].

However, despite these important contributions, several critical gaps remain insufficiently addressed. First, previous reviews largely focused on conventional scaffold engineering or material preparation strategies, while emerging applications of agarose in immunomodulation [35,36], biosensing [37], molecular diagnostics [38], cryopreservation [29], and intelligent biofabrication [39] have not been systematically discussed. Second, the structure–property–function relationship of agarose across diverse biomedical scenarios remains fragmented, particularly regarding how gelation behavior, mechanical tunability, porosity, and biointerface characteristics govern translational performance. Third, limited attention has been paid to the clinical translation potential of agarose biomaterials, including sterilization, large-scale manufacturing, regulatory considerations, and long-term biosafety.

Therefore, this review aims to provide a comprehensive and critical overview of agarose-based biomaterials, covering their structural characteristics, rheological properties, modification strategies, and emerging biomedical applications. Particular emphasis is placed on recent advances in immunomodulation, tissue repair, diagnostics, cryopreservation, and translational medicine. In addition, current clinical status, regulatory considerations, translational barriers, and future perspectives are critically discussed to clarify the opportunities and remaining challenges for the clinical development of agarose-based systems. To enable a critical comparison across these application areas, this review examines not only the reported performance of agarose-containing systems but also the specific function of agarose within each formulation, the contribution of additional components, the maturity of the supporting evidence, and the barriers to translation. Where component-matched controls are unavailable, effects observed in multicomponent systems are interpreted as formulation-level outcomes rather than as intrinsic properties of agarose.

A qualitative survey of studies published during the past five years highlights several active themes, including immunomodulatory hydrogels [40,41], 3D biofabrication [39,42,43,44], microphysiological systems [45,46], and cryopreservation and cell-protection strategies [29,47,48,49]. Single-cell methods are also beginning to provide higher-resolution assessment of cell–material responses [50].

**Figure 1:**
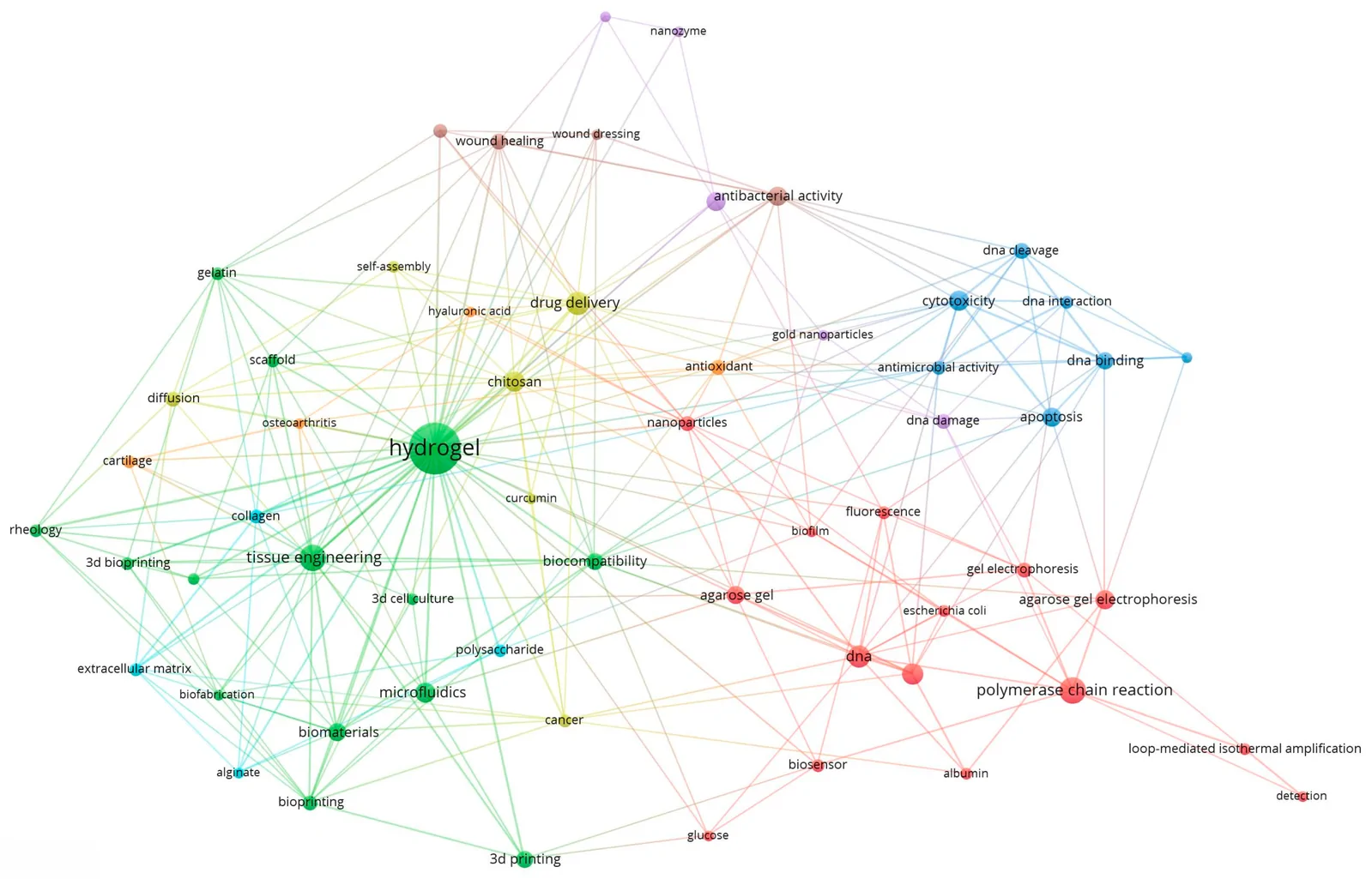
Co-occurrence network of author keywords in agarose-related biomedical research published between 2021 and 2025. Node size represents the frequency of keyword occurrence, node color indicates the corresponding thematic cluster, and connecting lines show co-occurrence relationships between keywords.

To identify recent research trends, 2910 research articles published between 2021 and 2025 were retrieved from the Web of Science Core Collection on 31 August 2026. The search combined “agarose” with terms related to biomaterials, tissue engineering, drug delivery, diagnostics, immunomodulation, and cryopreservation. A co-occurrence analysis of author keywords was performed using VOSviewer 1.6.20 with full counting. After synonymous terms were merged and general terms were removed, 59 keywords occurring at least 11 times were grouped into eight clusters (Figure 1). “Hydrogel” formed the central node, linking major topics in tissue engineering, drug delivery, biofabrication, biological evaluation, and diagnostic applications.

## 2. Agarose Chemistry and Properties

Agarose gelation is commonly described in three phases: induction, gelation, and pseudo-equilibrium. On cooling, agarose chains undergo coil-to-helix transition, followed by aggregation of helical domains into junction zones; hydrogen-bond-mediated physical association is central to network formation [51,52]. In contrast to polysaccharides that often require purpose-designed chemical crosslinking [53], agarose can form a network by physical gelation without covalent crosslinkers [51,52,54].

Agarose gel is an elastic and turbid material formed by the natural non-ionic polysaccharide agarose in aqueous solution. Fibrous micelles formed by agarose molecules act as crystalline junction zones, interconnected by polymer chains to construct a three-dimensional network. The resulting gel can stably retain water in its structure and maintain a specific morphology. Its elasticity originates from the entropic elasticity of agarose molecular chains connecting the crystalline junction zones. Its three-dimensional network structure resembles the extracellular matrix (ECM), providing a simulated microenvironment for biology-related research [55].

**Figure 2:**
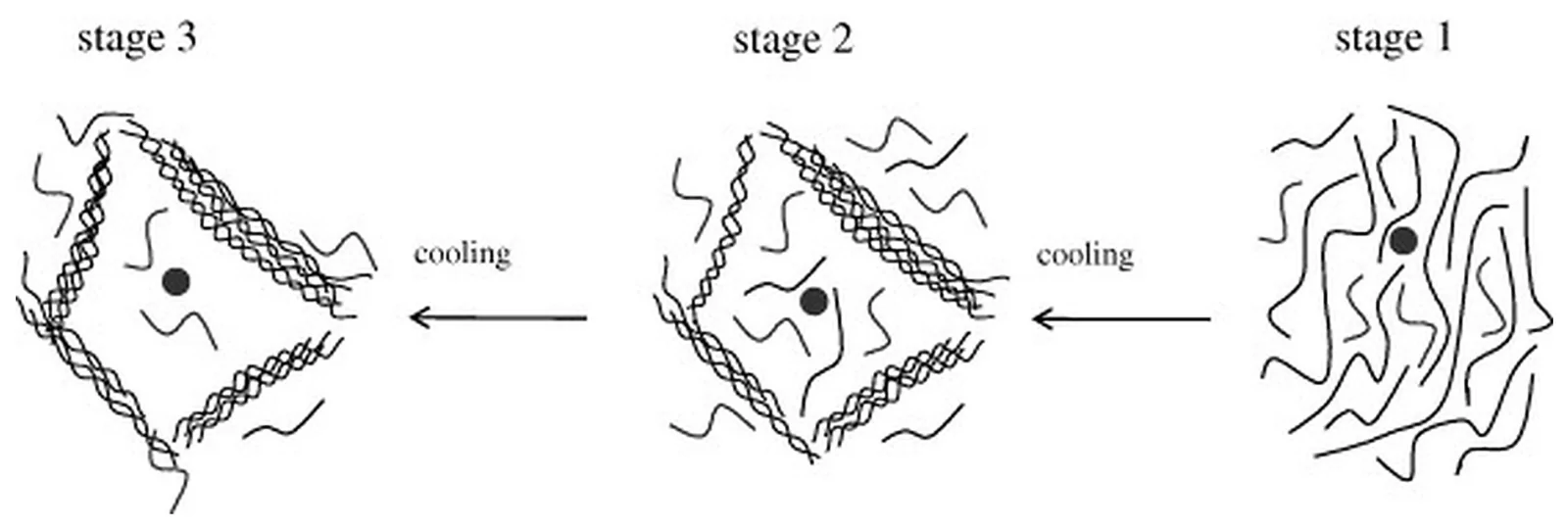
Schematic illustration of the microscopic environment evolution of an encapsulated guest during the three-stage sol–gel transition of agarose upon cooling (induction, gelation, and pseudo-equilibrium). Lines represent agarose polymer chains, and the closed sphere represents the encapsulated guest. Reprinted with permission from [52].

These properties support applications that bridge research and industry, including native and analytical electrophoresis [56,57,58,59], affinity chromatography and protein purification [60,61], microbial-cell encapsulation [62,63], diagnostic biosensors [64,65], controlled drug delivery [20,21,22], organoid and microphysiological platforms [45,66], cosmetic rheology modification [67], and bone-scaffold fabrication [68]. Additional uses illustrate the breadth of the platform: agarose fibers and humidity sensors [16,69], protein-crystallization media [70], spheroid enrichment [71], exosome-related affinity workflows [61,72], light- and humidity-responsive actuation [73], transparent tissue phantoms for electrode insertion [74,75], and agarose supports for enzyme immobilization [76,77]. The microscopic evolution of the agarose network during cooling and gel formation is schematically illustrated in Figure 2.

## 3. Agarose in Biomedicine

### 3.1. Separation and Detection of Nucleic Acids and Proteins

Disease diagnosis, therapeutic-response assessment, and prognosis increasingly rely on the combined measurement of protein and nucleic-acid biomarkers [78,79,80]. Because heterogeneous diseases are not always captured by a single marker, multiplex and multimodal assays are an important direction for in vitro diagnostics and point-of-care testing, spanning conventional immunoassays, chemiluminescent formats, and nucleic-acid/hydrogel systems [79,80,81,82].

Most in vitro diagnostic workflows rely on nucleic-acid amplification or immunoassays, while multiplex analysis is increasingly important for improving diagnostic specificity [79,80,82]. Combined detection of protein and nucleic-acid biomarkers may be especially useful for complex conditions such as sepsis [83,84].

By changing agarose concentration and processing conditions, pore size can be tuned for biomolecular separation. Agarose also provides an optically compatible, porous matrix for fluorescence and colorimetric readout, and can be integrated into electrophoretic and sensing platforms [56,57,65].

Sepsis is a life-threatening syndrome involving systemic and microcirculatory dysfunction and a major global health burden [85,86,87]. Circulating cell-free DNA (cfDNA) and histones are increasingly investigated as complementary biomarkers of host injury, severity, and outcome [83,84,88].

Shahriari and Selvaganapathy [89] developed an agarose-gated microfluidic device for the simultaneous fluorescence detection of a 150 bp DNA fragment used as a cfDNA surrogate and histones in plasma. Dehydrated agarose gates containing immobilized dyes rehydrate on sample loading, enabling on-chip staining and spatial separation in less than 20 min using 30 μL of plasma. The assay was evaluated using DNA and histones spiked into plasma at concentrations selected to represent healthy individuals, sepsis survivors, and non-survivors; clinical patient samples were not tested.

**Figure 3:**
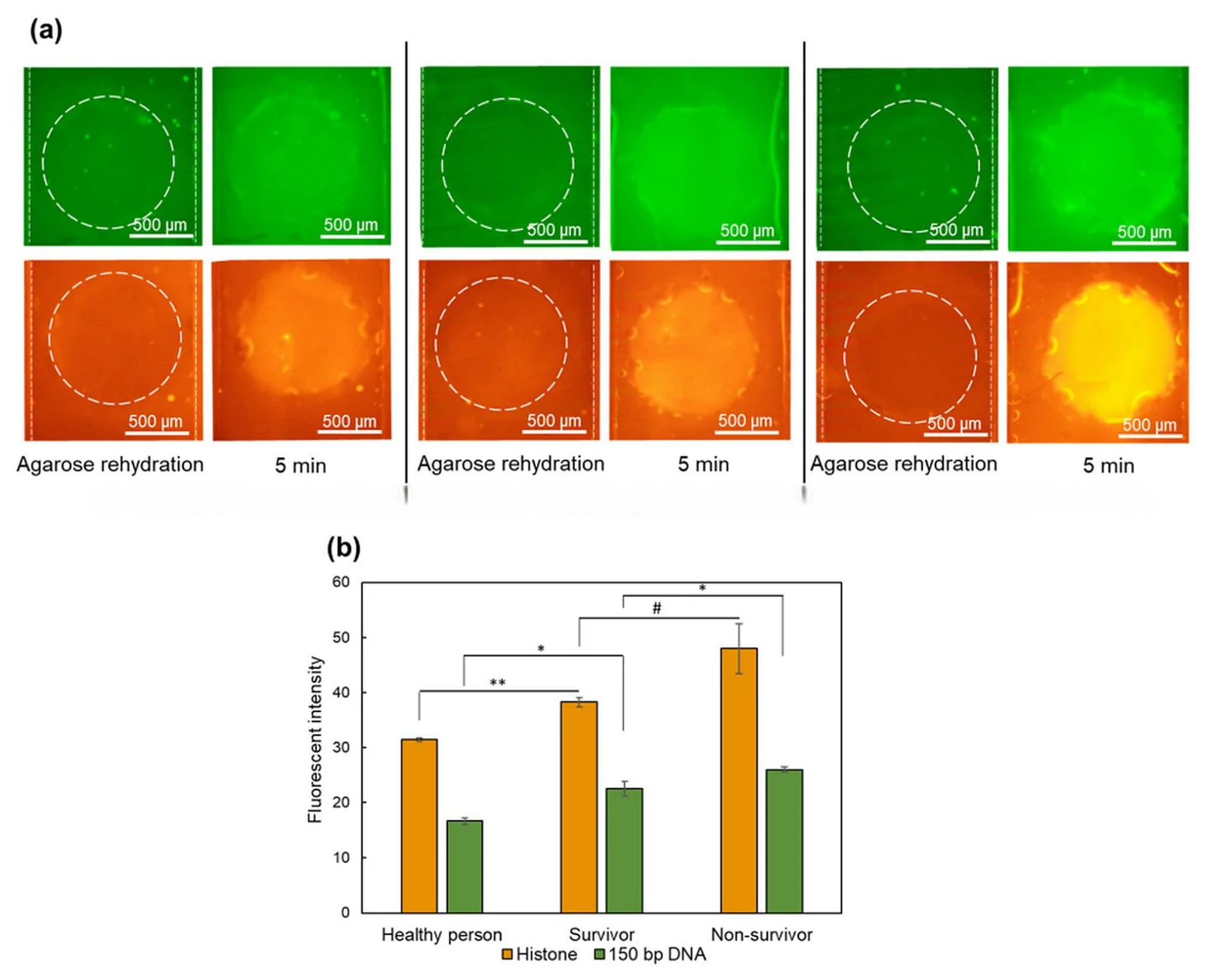
Proof-of-concept detection of a cell-free DNA (cfDNA) surrogate and histones in spiked plasma using the fully integrated microfluidic device. (**a**) Fluorescence images obtained from plasma samples prepared to represent biomarker concentrations associated with healthy individuals, sepsis survivors, and non-survivors; these conditions did not correspond to actual clinical patient groups (top row: 150-base-pair (bp) DNA used as a cfDNA surrogate; bottom row: histones). (**b**) Quantitative fluorescence intensities of DNA (green bars) and histones (orange bars) under the three simulated conditions. Dashed circles indicate the agarose-gate regions used for fluorescence visualization. Statistical significance was calculated using a two-tailed *t*-test (* *p* < 0.05, ** *p* < 0.01, and # *p* < 0.1; N = 3). Reprinted with permission from [89].

Compared with LC–MS/MS-based histone analysis [88], the agarose-gated device [89] offers a faster workflow, smaller sample requirement, and simpler instrumentation. However, the fluorescence assay does not distinguish individual histone species and was evaluated using spiked rather than clinical plasma, whereas the LC–MS/MS method was applied to plasma samples from critically ill patients [88]. The agarose-gated device should therefore be regarded as an analytical proof of concept rather than a clinically validated diagnostic platform. Testing with patient samples and broader analytical validation are still required before point-of-care use. Paper-based microfluidic fabrication methods provide alternative low-cost device-manufacturing routes [90,91], but do not replace the analytical and clinical validation required for the agarose-gated assay.Representative proof-of-concept results obtained with this integrated microfluidic device are shown in Figure 3.

### 3.2. Controlled Drug Release and Delivery

Unlike ionic polysaccharides, agarose forms a salt-independent, thermoreversible physical network. Its pore architecture and diffusion behavior can be adjusted through concentration, thermal history, and formulation; however, limited intrinsic bioactivity often necessitates blending or functionalization for therapeutic applications [31,92]. In unmodified agarose gels, drug release is governed mainly by diffusion through the hydrated network. Agarose therefore generally acts as a structural matrix or diffusion barrier rather than as an intrinsically stimulus-responsive release regulator.

Agarose-based materials have been investigated as biocarriers because of their biocompatibility, tunable porous structure, gelation behavior, and formulation flexibility [20,21,22,92]. Reported release strategies include passive sustained release and pH-, magnetic-, or acoustic-responsive delivery. These two modes should be distinguished: passive sustained release generally reflects diffusion through the agarose network, whereas stimulus-responsive behavior in agarose-containing composites is usually introduced by an added polymer, nanoparticle, or functional group. The wider polysaccharide-carrier literature includes chitosan, cellulose, hydroxypropyl methylcellulose, and marine-polysaccharide systems [93,94,95,96,97,98,99,100,101,102]; these provide useful comparators but should not be used as direct evidence for agarose-specific performance. Likewise, general pH-, redox-, and mechanically active hydrogel platforms illustrate the wider stimuli-responsive design space [103,104,105,106].

Agarose-specific examples extend to photocontrolled double networks [107], porous calcium-phosphate/agarose composites [108], electrically switchable deep-eutectic-solvent/agarose gels [109], RGD-modified agarose microcapsules [110], and sulfated or phosphorylated agarose for FGF-2 release [111]. In these systems, the observed function may arise from modification of the agarose phase itself or from the incorporated functional component. Accordingly, the contribution of agarose should be distinguished from that of the responsive polymer, ligand, or nanoparticle. Encapsulation and release performance vary substantially with the drug, formulation, loading method, and test conditions; therefore, formulation-specific metrics are more reliable than a single generalized efficiency range. Table 1 summarizes these systems at the formulation level and identifies the specific role attributed to agarose.

Philip et al. [112] developed an agarose–poly(acrylic acid) hybrid hydrogel as a pressure-sensitive adhesive platform for gastrointestinal ibuprofen delivery. Agarose formed the thermoreversible structural phase, whereas poly(acrylic acid) provided most of the pH-dependent swelling response. The study evaluated swelling, mechanical properties, and in vitro drug release under different pH conditions.

**Figure 4:**
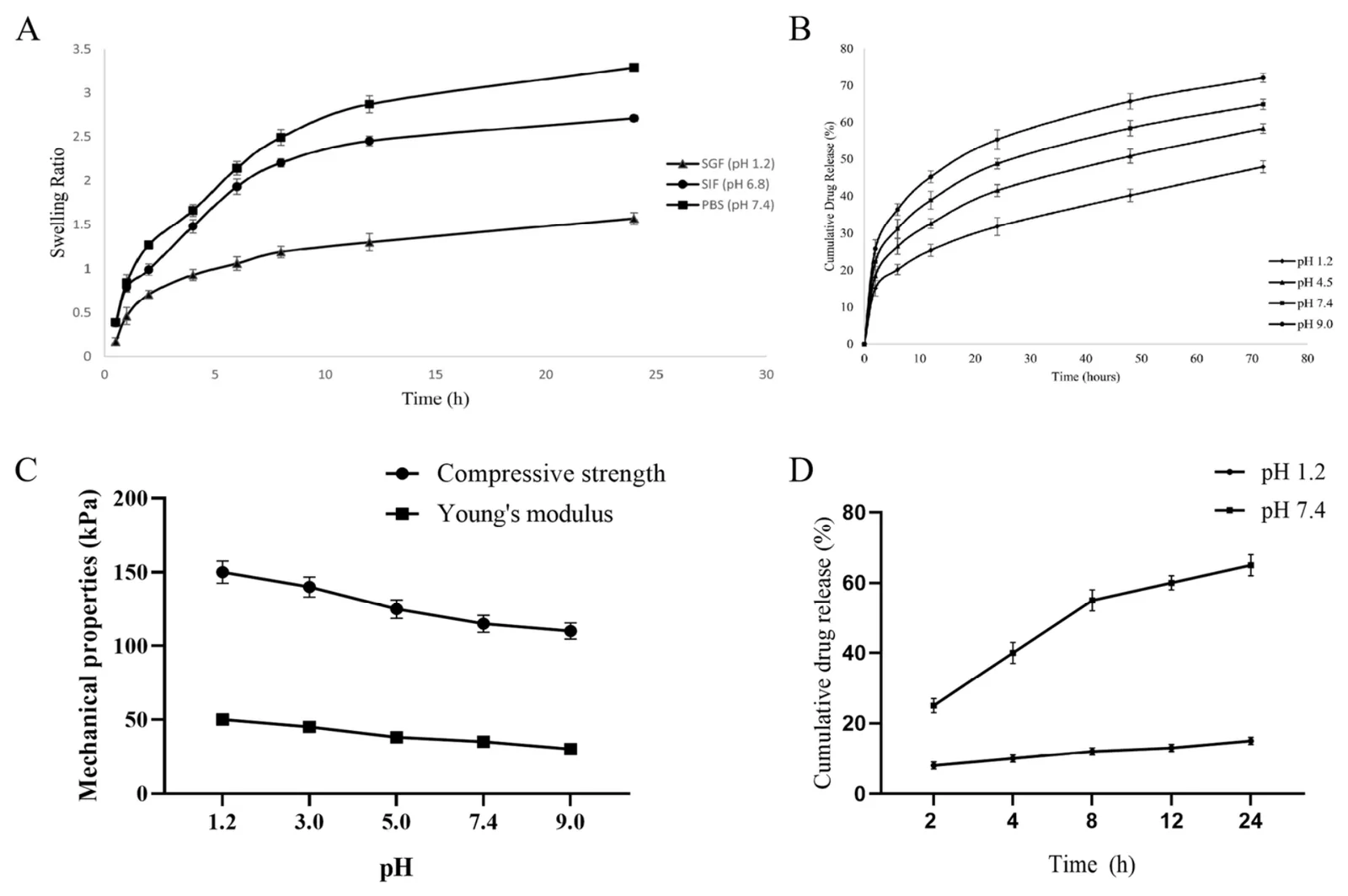
pH-dependent swelling, mechanical properties, and in vitro ibuprofen release of the agarose–poly(acrylic acid) hybrid hydrogel. (**A**) Swelling ratios in simulated gastric fluid (SGF, pH 1.2), simulated intestinal fluid (SIF, pH 6.8), and phosphate-buffered saline (PBS, pH 7.4) over 24 h. (**B**) Cumulative ibuprofen release at pH 1.2, 4.5, 7.4, and 9.0 over 72 h. (**C**) Compressive strength and Young’s modulus measured from pH 1.2 to 9.0. (**D**) Comparison of cumulative release at pH 1.2 and 7.4 over 24 h. Reprinted with permission from [112].

As shown in Figure 4, the hydrogel exhibited pH-dependent swelling, mechanical properties, and ibuprofen release. The swelling ratio increased from 1.58 ± 0.09 at pH 1.2 to 3.29 ± 0.12 at pH 7.4, while both compressive strength and Young’s modulus decreased as the pH increased. After 24 h, approximately 15% of the incorporated ibuprofen was released at pH 1.2, compared with approximately 65% at pH 7.4 [112]. These results demonstrate pH-dependent release from the agarose–poly(acrylic acid) formulation under in vitro conditions, but they do not directly establish gastric retention, reduced gastric irritation, or in vivo intestinal targeting.

**Table 1:** Agarose-containing drug-delivery systems: formulation, experimental model, release behavior, and the specific contribution of agarose.

Formulation and Reported Agarose Content	Cargo	Release Test and Study Model	Release Behavior and Controlling Component	Specific Role of Agarose
CHAp/AG; 1 wt% AG [113]	AMX; 5-FU	In vitro release: PBS, pH 7.4, 37 °C, 100 rpm; cell model: MG-63; antibacterial model: *E. coli*, *S. aureus*, and *S. epidermidis*	Sustained release governed by porosity and drug–carrier affinity	Coating and diffusion barrier
AG/Carbomer (crosslinked PAA); 1 g AG and 2 g Carbomer/100 mL [112]	Ibuprofen	In vitro gastrointestinal release: pH 1.2, 6.8, and 7.4, 37 °C, 72 h; no cell or animal model	<15% at pH 1.2 and 64.9% at pH 7.4 after 24 h; PAA controlled the pH response	Thermoreversible scaffold
CMC/AG/CeO_2_; 0.4 g AG/40 mL [114]	Quercetin	In vitro release: pH 5.4/7.4, 37 °C, 96 h; cell models: HepG2 and L929	98%/64%; controlled mainly by CMC and CeO_2_	Mechanical support
β-CD-functionalized AG; 2% *w*/*v* [115]	BSA; DOX	In vitro release: PB, pH 7.4, 37 °C, 24 h; cell models: HEK-293 and HeLa	Rapid BSA release; sustained DOX release through β-CD complexation	Gel-forming matrix
AG/CMC/MoS_2_ double emulsion [116]	Curcumin	In vitro release: pH 5.4/7.4, 96 h; cell models: A549 and L929	pH-dependent release; controlled mainly by CMC/MoS_2_	Structural matrix
AG/Carbopol 934P; optimized formulation F9 contained 0.3% *w*/*v* AG [117]	Benzocaine; tibezonium iodide	In vitro release: 3 h; ex vivo buccal-mucosa testing; mucoadhesive residence-time and local-tolerance evaluation in healthy volunteers	Approximately 99% release within 3 h; Carbopol improved mucoadhesion	Gel-forming and mucoadhesive matrix
PDA/Py-CNC/AG; DA/AG 8%, Py-CNC/AG 12.5% [118]	Paclitaxel	In vitro release: pH 5.5/7.4, 8 days; cell models: MCF-7 and BMEC	86.3%/10.2%; controlled by PDA and the IPN	Component of IPN
Pluronic/PMAA/AG; AG 0.4–1.2 wt% [119]	Cyclophosphamide	In vitro gastrointestinal release: pH 1.2/7.4, 37 °C, 24 h; in vivo safety model: rabbits	pH-dependent release controlled mainly by PMAA	Supporting network
CS/AG/g-C_3_N_4_; AG 1% *w*/*v* [120]	Curcumin	In vitro release: pH 5.4/7.4, 37 °C, 96 h; cell model: MCF-7	Higher acidic release; controlled by CS and g-C_3_N_4_	Matrix stabilizer
CS/AG/γ-Al_2_O_3_; 1 g AG/100 mL [121]	5-FU	In vitro release: pH 5.4/7.4, 37 °C, 96 h; cell model: MCF-7	94%/49% at 48 h; controlled by CS and the double emulsion	Hydrogel framework
AG/PVA-β-CD; 14 mg AG/2.5 mL [122]	IBU-Na; IBU	In vitro release: PBS, pH 7.4, 37 °C, static, 7 h; no cell or animal model	Fickian diffusion; β-CD controlled hydrophobic IBU	Physical gel network
CS/AG/GO; AG 1% *w*/*v* [123]	5-FU	In vitro release: pH 5.4/7.4, 37 °C, 96 h; cell model: MCF-7	96.0%/78.9%; controlled mainly by CS/GO	Supporting matrix
CS/Pluronic/AG with alginate beads; AG 1% *w*/*v* [124]	Tetracycline	In vitro release: pH 5.8–7.4, 37 °C, 48 h; cell model: HFF2; hemocompatibility test	Sustained release controlled mainly by alginate beads	Semi-IPN matrix
Freeze-dried AG/Eudragit; 30 mg AG/unit [20]	Theophylline	In vitro gastrointestinal release: sequential pH 1.5 → 4.0 → 6.8, 37 °C, 8 h; no cell or animal model	RL-PO produced sustained, pH-independent release	Rehydratable porous matrix
AG/GQDs/α-Fe_2_O_3_; 0.5 g AG/25 mL [125]	Quercetin	In vitro release: pH 5.4/7.4, 37 °C, 96 h; cell models: HepG2 and L929	96%/56%; controlled mainly by GQDs and matrix interactions	Carrier matrix
Nanoassembled AG microspheres [126]	DOX; irinotecan	In vitro release: >120 h; application model: TACE-oriented embolic microspheres	Sustained release; nanoparticles used mainly for imaging	Drug reservoir and embolic scaffold
AG/CS/MNPs-OA; optimized AG/CS 80:20 [127]	DOX	In vitro release: pH 4.5/7.4, 20 rpm, 4 days; cell model: MCF-7	Higher acidic release; controlled mainly by CS	Porous mechanical scaffold

Table 1 should be interpreted at the formulation level rather than as evidence that each response arises from agarose alone. Study models are identified as in vitro, ex vivo, in vivo, or analytical/device, and unavailable experimental details were not inferred. In responsive composites, agarose generally provides the hydrated matrix, pore structure, or mechanical support, whereas pH-, magnetic-, or electrically responsive behavior may originate primarily from the added functional component. The wireless SAW study [128] was excluded because agarose served as a synthetic skin surrogate rather than as the drug-delivery carrier.

Abbreviations: 5-FU, 5-fluorouracil; AG, agarose; AMX, amoxicillin; BSA, bovine serum albumin; β-CD, β-cyclodextrin; CeO_2_, cerium dioxide; CHAp, carbonated hydroxyapatite; CMC, carboxymethyl cellulose; CS, chitosan; DA, dopamine; DOX, doxorubicin; GO, graphene oxide; GQDs, graphene quantum dots; g-C_3_N_4_, graphitic carbon nitride; IBU, ibuprofen; IBU-Na, sodium ibuprofen; IPN, interpenetrating polymer network; MNPs-OA, oleic-acid-coated magnetic nanoparticles; MoS_2_, molybdenum disulfide; PAA, poly(acrylic acid); PB, phosphate buffer; PBS, phosphate-buffered saline; PDA, polydopamine; PMAA, poly(methacrylic acid); PVA, poly(vinyl alcohol); Py-CNC, pyrene-labeled cellulose nanocrystals; RL-PO, Eudragit RL PO; semi-IPN, semi-interpenetrating polymer network; TACE, transarterial chemoembolization; α-Fe_2_O_3_, alpha-iron(III) oxide; γ-Al_2_O_3_, gamma-alumina. “pH-responsive” describes the complete formulation and does not imply intrinsic pH responsiveness of agarose. Magnetic particles were considered targeting or imaging components unless field-triggered release was demonstrated.

### 3.3. Tissue Repair

Agarose-based systems have been explored for bone, cartilage, skin, neural, osteochondral, and infected-bone repair [14,18,68,129,130,131,132,133]. In most cases, agarose serves as a hydrated structural matrix and is combined with gelatin or GelMA, hyaluronic acid, hydroxyapatite, conductive polymers, growth factors, or antimicrobial components to compensate for its limited intrinsic cell adhesiveness and bioactivity. Agarose–hyaluronan wound scaffolds and BCP-CSD-agarose microspheres provide further examples of soft- and hard-tissue formulations [134,135]. The available evidence ranges from physicochemical characterization and in vitro cell assays to a smaller number of animal studies; therefore, the reported outcomes should be interpreted in relation to the complete formulation and the model used rather than as general repair functions of agarose. These studies sit within a wider cartilage-engineering literature that includes scaffold-free printing, injectable gelatin hydrogels, and scaffold-degradation effects on matrix development [2,42,136,137]. Representative agarose formulations, research models, and their proposed functions are summarized in Table 2.

One reported agarose–gelatine–hydroxyapatite–minocycline nanocomposite exhibited a compressive modulus in the range of approximately 15–25 MPa under the study’s test conditions [142]. This mechanical response reflects the complete mineralized composite rather than agarose alone, because gelatin and hydroxyapatite also contribute to network reinforcement and load transfer. This range overlaps values reported for articular cartilage and some osteochondral scaffolds [143,144,145], but direct comparisons should be interpreted cautiously because specimen geometry, strain rate, hydration, and testing protocol differ among studies. The result supports further mechanical evaluation; it does not by itself establish equivalence to native cartilage or demonstrate functional tissue repair.

**Figure 5:**
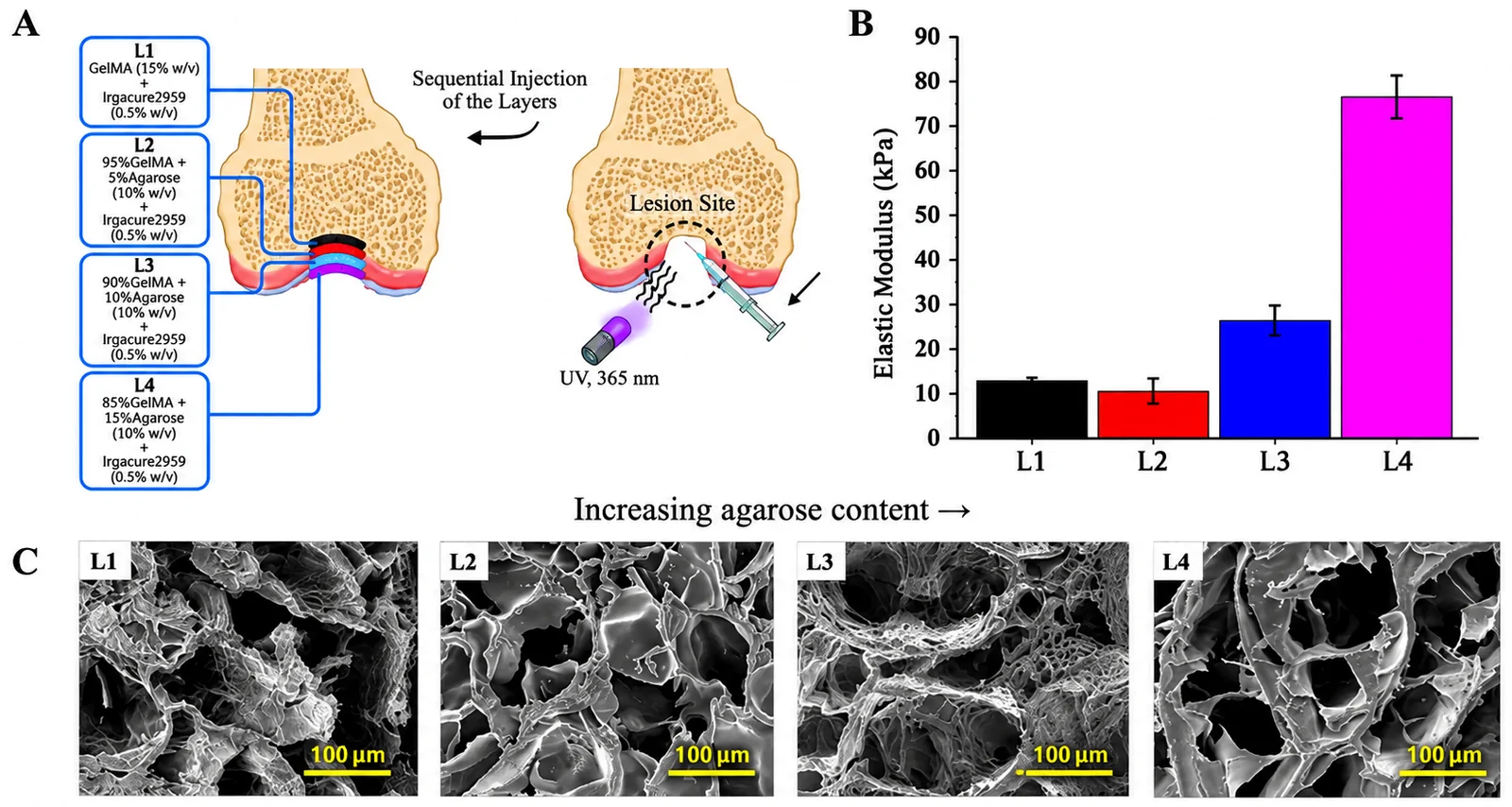
Gradient gelatin methacryloyl (GelMA)/agarose hydrogel scaffold for osteochondral tissue engineering and its structural and mechanical characteristics. (**A**) Schematic illustration of the four-layer injectable GelMA/agarose scaffold for osteochondral defect repair through sequential injection and in situ photocrosslinking. (**B**) Compressive modulus of different hydrogel layers, demonstrating enhanced mechanical strength with increasing agarose content. (**C**) Scanning electron microscopy (SEM) images of scaffold layers 1–4 (L1–L4) showing interconnected porous architectures and gradient microstructural variations associated with the different GelMA/agarose compositions. Reprinted with permission from [14].

Mokhtarzade et al. [14] developed a four-layer injectable GelMA/agarose gradient hydrogel scaffold, in which the agarose content was gradually increased to achieve a continuous transition from the subchondral bone layer to the cartilage layer (Figure 5A). In this system, GelMA mainly provided photocrosslinking capability and cell-adhesive motifs, whereas agarose contributed to the formation of a cartilage-like microenvironment and enhanced structural stability through its thermogelation properties. Moreover, the scaffold could be sequentially injected and photocrosslinked in situ, supporting the fabrication of a spatially graded construct. However, injectability and in situ gelation were demonstrated as material-processing properties, whereas minimally invasive repair of an osteochondral defect was not evaluated in an animal model.

Further investigations demonstrated that the incorporation of agarose was associated with changes in the microstructural characteristics of the scaffold but also significantly enhanced its mechanical properties. As the agarose content increased, the compressive modulus of the hydrogel was markedly elevated (Figure 5B), indicating that agarose effectively reinforced the hydrogel network under the reported test conditions. However, these measurements do not establish equivalence to the load-bearing properties of native cartilage tissue. In addition, SEM images revealed interconnected porous architectures within all scaffold layers (Figure 5C). Gradient variations in pore morphology were observed with increasing agarose content, which may be relevant to cell infiltration, nutrient transport, and extracellular matrix deposition. However, these biological functions were not directly demonstrated by the SEM observations. The reported short-term cell-viability results support cytocompatibility, but longer-term cell-distribution and in vivo osteochondral-defect studies are required to determine whether the gradient architecture improves tissue integration and functional repair.

### 3.4. Immunomodulation

Immune regulation is increasingly recognized as a determinant of tissue repair and biomaterial integration [40,146]. Implanted materials can influence innate immune-cell recruitment, macrophage polarization, foreign-body responses, angiogenesis, and remodeling [146,147].

Agarose-based biomaterials offer a highly hydrated physical network, generally favorable biocompatibility, and low intrinsic cell adhesiveness [31,148,149]. Because agarose networks can be formed by physical gelation rather than covalent crosslinking [51,52,54], they can avoid residual chemical crosslinkers, although blended or chemically modified formulations require separate cytocompatibility assessment.

Agarose-based hydrogels can influence immune-cell behavior through their physical properties and incorporated cues [23,92,142]. In the zymosan/ι-carrageenan/agarose system reported by Venkatachalam et al. [41], the changes in TNF-α, CXCL10, TLR2, and M2-associated markers were measured after treating differentiated THP-1 macrophages with zymosan rather than with the complete hydrogel. These antitumoral, macrophage-associated effects should therefore be attributed primarily to zymosan. Agarose was used as a gelling agent to stabilize the composite network, while ι-carrageenan contributed to gel stability and swelling. Macrophage activation is better understood as a dynamic spectrum rather than a strict two-state system, although M1-like and M2-like labels remain useful shorthand for predominantly inflammatory and reparative phenotypes, respectively [92,150].

**Figure 6:**
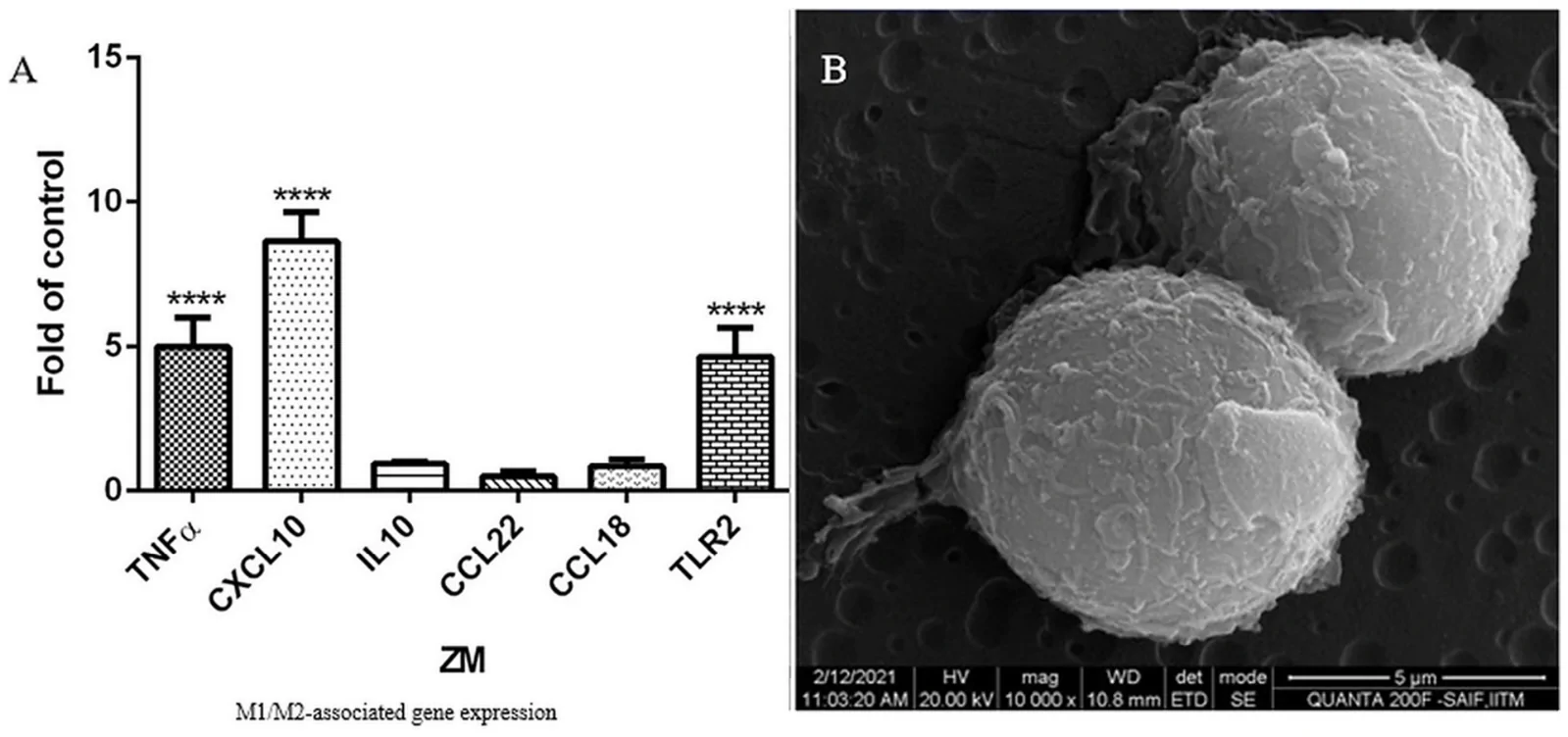
Component-level immunomodulatory responses and composite-hydrogel cell attachment relevant to agarose-containing systems. (**A**) Expression of classically activated (M1)-associated, alternatively activated (M2)-associated, and Toll-like receptor 2 (TLR2) genes in differentiated human THP-1 monocytic cells treated with zymosan; the complete hydrogel was not used in this assay. (**B**) Porous morphology and attachment of murine L929 fibroblasts observed for the zymosan/ι-carrageenan/agarose hydrogel. In the original panel labels, ZM denotes zymosan. Statistical significance was calculated using a two-tailed *t*-test (**** *p* < 0.0001). Reprinted with permission from [41].

The immunomodulatory behavior of agarose-based systems depends strongly on physicochemical properties such as stiffness, pore size, surface chemistry, and incorporated bioactive cues [146,151,152]. These parameters may influence macrophage adhesion and polarization; however, responses are formulation- and tissue-dependent and should not be generalized across all agarose hydrogels. The corresponding immunomodulatory responses and composite-hydrogel cell attachment are illustrated in Figure 6.

Agarose can also serve as a carrier for antibacterial agents and other bioactive components [153,154,155,156]. Incorporation may enable local retention or controlled presentation, but the release kinetics, dose, degradation behavior, and host response must be validated for each composite.

### 3.5. Cryopreservation and Cell Protection

Conventional mammalian cell cryopreservation commonly relies on DMSO-based freezing media, typically containing 5–10% DMSO with serum or serum-free basal media [157]. DMSO effectively penetrates cell membranes and reduces intracellular ice formation, thereby maintaining high post-thaw viability [157]. However, its cytotoxicity, osmotic stress, and potential adverse effects in cell therapy products have stimulated the development of DMSO-free cryopreservation strategies [158]. Commercial cryopreservation media provide standardized formulations, but their performance depends on cell type, DMSO concentration, and the post-thaw assessment time [159,160,161]. In contrast, trehalose is a non-permeating cryoprotectant with low toxicity and strong water-replacement and vitrification effects, although trehalose alone usually provides insufficient protection for mammalian cells due to limited membrane permeability [158,162].

To reduce reliance on membrane-penetrating cryoprotectants, Wang et al. [29] investigated mammalian-cell encapsulation in 0.75% agarose hydrogels containing trehalose. The agarose matrix reduced ice-recrystallization-related damage, and the optimized agarose/trehalose condition produced post-thaw viability comparable to 10% DMSO in the reported cell system, although post-thaw proliferation remained lower.

**Figure 7:**
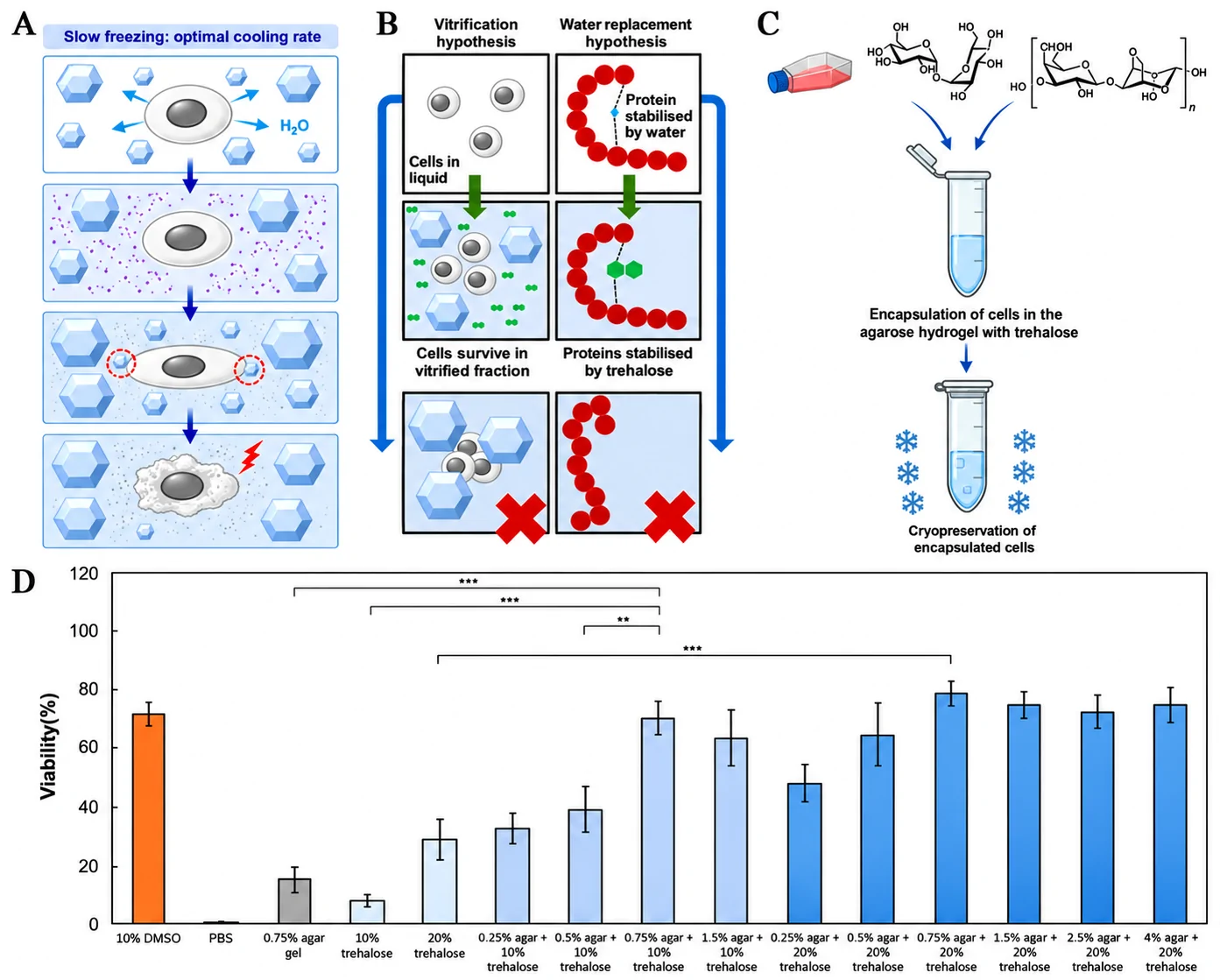
Mechanisms and strategies of agarose/trehalose hydrogel-assisted cryopreservation. (**A**) Cryoinjury pathways during conventional slow freezing, including osmotic dehydration, vitrification of the unfrozen fraction, ice recrystallization during warming, and subsequent membrane damage. (**B**) Cryoprotective mechanisms of trehalose based on the vitrification and water replacement hypotheses. (**C**) Encapsulation of mammalian cells within agarose hydrogels containing trehalose for dimethyl sulfoxide (DMSO)-free cryopreservation. (**D**) Post-thaw viability of cells preserved using different cryoprotective systems, showing immediate post-thaw viability comparable to conventional 10% DMSO in the reported cell system; equivalent post-thaw proliferation was not demonstrated. In panel (**B**), gray circles represent cells, and red cross symbols indicate the inhibition of cryoinjury-related processes. Statistical significance is indicated as (** *p* < 0.01 and *** *p* < 0.001). Reprinted with permission from [29].

Figure 7 and Table 3 collectively summarize the mechanisms and performance of agarose/trehalose hydrogel-assisted cryopreservation systems. As illustrated in Figure 7, conventional slow freezing may induce multiple forms of cryoinjury, including osmotic dehydration, intracellular ice formation, ice recrystallization during thawing, and membrane damage. Trehalose exerts cryoprotective effects mainly through the vitrification and water replacement hypotheses, thereby stabilizing proteins and cellular structures during freezing. Furthermore, encapsulation of mammalian cells within agarose hydrogels provides a confined microenvironment that restricts ice crystal growth and reduces free water mobility, ultimately improving post-thaw cellular recovery [29].

Table 3 compares conventional and hydrogel-assisted cryopreservation strategies. Ten percent DMSO is a widely used benchmark. Commercial media offer standardized formulations, but post-thaw outcomes remain cell- and assay-dependent [159,160,161]. Trehalose alone has limited efficacy for many mammalian cells because of poor membrane permeability. In the reported study, agarose/trehalose encapsulation achieved post-thaw viability comparable to 10% DMSO while avoiding a membrane-penetrating cryoprotectant; however, lower post-thaw proliferation and the absence of broad cell-type and scale-up validation remain important limitations [29].

## 4. Clinical Translation and Current Applications

Compared with alginate, which has established clinical use in wound-dressing products [163,164], agarose-based implantable and regenerative systems remain predominantly preclinical [31,165,166,167]. Recent alginate and chitosan/alginate dressing studies illustrate 3D printing, metal-nanoparticle incorporation, and metal–organic-framework/curcumin composites [168,169,170]; chitosan- and glucan-based dressing literature provides additional benchmarks [101,171]. Agarose nevertheless has long-standing commercial value in electrophoresis, bioseparation, and laboratory diagnostics. Table 4 therefore distinguishes analytical-market maturity from clinical validation of implantable biomaterials.

Agarose is widely established as a matrix for molecular separation and bioseparation [56,57,58,60,61]. By contrast, therapeutic delivery, injectable scaffolds, and regenerative implants remain largely at the preclinical or early translational stage [31,165,166,167]. It is therefore more accurate to describe agarose as a well-established analytical and bioseparation material than as a clinically validated implantable biomaterial.

Agarose hydrogels, nanocomposites, microspheres, and injectable systems have been extensively investigated for the controlled delivery of anticancer drugs, antibiotics, anti-inflammatory agents, and bioactive molecules [20,112,113,114,115,116,128]. In addition, owing to their extracellular matrix-like three-dimensional architecture, injectability, and bioprintability, agarose hydrogels have been widely explored for cartilage repair, bone regeneration, osteochondral engineering, skin repair, neural regeneration, and cell delivery applications [14,18,130,131,132,140,141]. Composite systems incorporating hydroxyapatite, gelatin, hyaluronic acid, conductive polymers, or bioactive nanoparticles have further expanded the biomedical applicability of agarose-based scaffolds.

Another emerging application is cell encapsulation and preservation. Mild processing and a porous hydrogel network make agarose useful for microbial and mammalian cell immobilization, organoid handling, and cryoprotection [29,47,48,63,66]. Translation will require validation across cell types, standardized freezing and warming protocols, and long-term functional assessment after recovery. Overall, ex vivo analytical and cell-processing applications are more readily translatable, whereas implantable systems face additional requirements for biodegradation, sterilization, manufacturing reproducibility, and long-term safety.

A cross-domain comparison of the roles, evidence maturity, advantages, and translational priorities of agarose-based biomedical systems is provided in Table 5.

## 5. Challenges and Future Perspectives

Despite the rapid development of agarose-based biomaterials in drug delivery, tissue engineering, diagnostics, and cryopreservation, several critical challenges still hinder their broader clinical translation. One of the major limitations of agarose is its poor biodegradability in mammals, since mammalian tissues generally lack agarase enzymes capable of efficiently degrading agarose networks [32,172]. As a result, long-term implantation may lead to prolonged material persistence and insufficient tissue remodeling. In addition, native agarose exhibits relatively limited intrinsic bioactivity and lacks cell-adhesive motifs, which may restrict cell attachment, proliferation, and tissue integration [31]. Although composite systems incorporating gelatin, collagen, hyaluronic acid, peptides, or bioactive nanoparticles can partially overcome these limitations, optimization of bioactivity while maintaining structural stability remains an important challenge [32]. Adhesive-peptide functionalization and blending with GelMA or collagen directly introduce cell-binding sites, whereas hyaluronic acid improves hydration and matrix-like properties and mineral fillers are particularly relevant to bone-oriented scaffolds. Controlled chemical functionalization may provide more reproducible bioactivity than increasingly complex multicomponent formulations. However, these strategies do not necessarily resolve the slow degradation of agarose; they may improve cellular or mechanical performance while leaving long-term matrix persistence largely unchanged.

A second challenge is balancing mechanical properties, injectability, and printability. Increasing agarose concentration can improve rigidity and shape retention but may reduce cell migration, mass transport, or processability. Composite and shear-processed systems offer useful design routes, but should be benchmarked under application-relevant conditions [31,39,151].

From a translational perspective, large-scale manufacturing, sterilization, batch-to-batch reproducibility, and regulatory pathways remain insufficiently established for many therapeutic agarose systems [165,166,167]. Increasing compositional complexity can improve performance while also complicating characterization, manufacturing control, and safety evaluation [173,174].

Near-term work should prioritize standardized formulations, batch-release criteria, sterilization compatibility, and application-specific benchmarks for mechanical properties, transport, release, and cell response. Longer-term studies should develop controllable degradation strategies, evaluate remodeling and biosafety in relevant long-term animal models, and establish scalable manufacturing and regulatory pathways for clinically intended systems. Stimuli-responsive delivery, immune-instructive scaffolds, mechanically robust yet processable gels, and microphysiological platforms remain valuable research directions, but their translational benefits should be assessed against simpler formulations [39,40,45,165,166,167,175,176].

## Figures and Tables

**Table 2:** Representative agarose-based hydrogels and composite scaffolds for tissue engineering and regenerative medicine.

Formulation	Intended Application	Research Model/Evidence Level	Key Outcome/Controlling Component	Specific Role of Agarose
Agarose hydrogels and composites [34]	Bone tissue engineering	Review synthesis; no single model	Performance depends on modification or added bioactive components	Hydrated thermogelling matrix; limited intrinsic cell adhesion
Agarose/PEGDA/nHA [138]	Cartilage engineering	L929 cells in vitro; no animal model	nHA improved mechanics and cell viability	Printable structural network; PEGDA crosslinks and nHA reinforces
PMP/Fe_3_O_4_@APNs/VAN-PLGA/agarose [130]	Infected bone-defect repair	Bacterial tests, rat BMSCs and rabbit tibial defects	VAN/microwave heating controlled antibacterial activity; phosphate cement supported osteogenesis	Encapsulated VAN-PLGA microspheres and integrated the delivery phase
Agarose/hydroxyapatite aerogel [68]	Bone scaffold	Material and mechanical tests only	HA supplied the mineral phase and increased stiffness	Formed the porous aerogel matrix
GelMA/agarose gradient scaffold [14]	Osteochondral engineering	hADMSC cytotoxicity assay; no animal model	GelMA/agarose ratio controlled layer-dependent properties	Adjusted viscosity, gelation and mechanics; GelMA provided photocrosslinking
Activated agarose-EGF [139]	Growth-factor presentation	Human dermal fibroblasts in vitro	Immobilized EGF retained proliferative activity	Immobilization carrier; EGF drove proliferation
DEX-PLGA/agarose implant [140]	Osteochondral repair	In vitro cartilage models and canine model	DEX-PLGA provided sustained chondroprotection	Retained microspheres at the repair site
Agarose/hyaluronic acid hydrogel [132]	Photoaged-skin repair	Fibroblasts, zebrafish and murine photoaging models	Hyaluronic acid enhanced hydration and dermal restoration	Crosslinker-free thermogelling network
Carboxylated agarose/SF/HA [141]	Tibial bone repair	MG63 cells and rat tibial defects	HA supported mineralization; SF slowed degradation	Reactive organic matrix supporting HA formation
Agarose/gelatin/PPy [131]	Spinal-cord repair	Neural stem cells and rat SCI model	PPy supplied conductivity and supported neuronal differentiation	Stabilized the injectable network and tuned modulus

Abbreviations: APNs, phytic acid dodecasodium-modified black phosphorus nanosheets; BMSCs, bone marrow-derived mesenchymal stem cells; DEX, dexamethasone; EGF, epidermal growth factor; Fe_3_O_4_, magnetite; GelMA, gelatin methacryloyl; HA, hydroxyapatite; hADMSC, human adipose-derived mesenchymal stem cell; nHA, nanohydroxyapatite; PEGDA, poly(ethylene glycol) diacrylate; PLGA, poly(lactic-co-glycolic acid); PMP, Fe_3_O_4_@APNs-loaded magnesium phosphate cement scaffold (sample designation used in Ref. [130]); PPy, polypyrrole; SCI, spinal cord injury; SF, silk fibroin; VAN, vancomycin.

**Table 3:** Comparison of conventional and hydrogel-assisted cryopreservation strategies for mammalian cells.

System	Main CPA	Reported Post-Thaw Outcome	Advantages	Limitations
Conventional DMSO freezing [157,159,160,161]	10% DMSO	Reference condition; cell-dependent	Simple and widely used	DMSO toxicity; handling constraints
Commercial DMSO-containing media [159,160,161]	Formulation-dependent DMSO	Cell- and assay-dependent	Standardized formulation	Product-specific performance
Trehalose only [29]	250 mM trehalose	~11% in the reported cell model	Low toxicity	Poor membrane permeability
Agarose/trehalose hydrogel [29]	0.75% agarose + 10–20% trehalose	Comparable to 10% DMSO immediately post-thaw	DMSO-free, reduced ice recrystallization	Lower proliferation after thawing

Abbreviations: CPA, cryoprotective agent; DMSO, dimethyl sulfoxide.

**Table 4:** Comparison of physicochemical properties and clinical translation potential between alginate- and agarose-based biomaterials.

Category	Alginate	Agarose
Natural source	Brown algae	Red algae
Gelation mechanism	Ionic crosslinking, commonly with Ca^2+^, rapid gelation under mild conditions	Thermoreversible physical gelation through heating and cooling
Biocompatibility	Generally favorable, but affected by purity, crosslinking, and formulation [163]	Generally favorable and low-immunogenic, but affected by purity, modification, and additives [31]
Intrinsic cell adhesion	Poor; often requires ligand modification or polymer blending	Poor; often requires chemical modification or composite formulation
Mechanical behavior	Soft; stability depends on ionic crosslinking and the physiological environment	Good shape retention; mechanically tunable but potentially brittle
Injectability	Widely explored through in situ ionic gelation	Feasible with appropriate thermal and rheological control [39]
Bioprinting	Widely explored; printability is commonly improved by blending or secondary crosslinking	Growing use in composite bioinks and support-bath systems; limited by its thermal processing window [34,39]
In vivo degradation	Composition- and formulation-dependent; ionic networks may lose stability under physiological conditions [163]	Limited in mammals; prolonged persistence remains a major concern [32]
Drug delivery	Established in some wound-care formats; other delivery systems remain mainly preclinical [163,164]	Controlled and stimulus-responsive systems remain predominantly preclinical [20,113]
Tissue engineering	Wound care, cartilage, and cell encapsulation; evidence ranges from clinical products to preclinical constructs [163]	Cartilage, bone, neural, and osteochondral repair; evidence remains predominantly preclinical [14,18,131,132]
Clinical maturity	Established wound-dressing products; maturity varies across other implantable applications [163,164]	Widely established in analytical use, but implantable systems remain mainly preclinical [56,57,58,59,60,61,165,166,167]
Representative products	KALTOSTAT^®^ wound dressing [164]	Electrophoresis gels and bioseparation media [56,57,58,60,61]
Key translational limitation	Limited mechanical durability and ionic stability in physiological environments	Limited mammalian degradability, low intrinsic bioactivity, and uncertain long-term in vivo fate [31,32,165,166,167]

**Table 5:** Cross-domain comparison of agarose-based biomedical applications.

Application Area	Role of Agarose	Evidence Maturity	Key Advantage	Main Barrier/Priority
Diagnostics	Separation, sensing, or microfluidic matrix	Established for laboratory separation; diagnostic devices remain proof-of-concept	Optical clarity and tunable pores	Larger cohorts and standardized device validation
Drug delivery	Matrix, diffusion barrier, or carrier	Mainly in vitro and preclinical	Tunable diffusion and compatibility with functional additives	Formulation-dependent release; requires in vivo pharmacokinetic and safety studies
Tissue repair	Structural scaffold or composite matrix	In vitro to preclinical animal studies	Thermogelation, porosity, and shape retention	Limited degradation and cell adhesion; requires long-term remodeling studies
Immunomodulation	Physical matrix or carrier for bioactive cues	Mainly in vitro proof-of-concept	Tunable physical properties and local cue retention	Effects often arise from added agents; requires component controls and in vivo validation
Cryopreservation	Encapsulation matrix with trehalose	Limited experimental proof-of-concept	Reduced ice damage without a membrane-penetrating CPA	Lower post-thaw proliferation; requires broader cell and scale-up validation

Abbreviation: CPA, cryoprotective agent.

## Data Availability

No new data were created or analyzed in this study. Data sharing is not applicable to this article.

## References

[1] Mozafari M., Ramedani A., Zhang Y.N., Mills D.K., Griesser H.J. (2016). Thin films for tissue engineering applications. Thin Film Coatings for Biomaterials and Biomedical Applications.

[2] Langer R., Vacanti J.P. (1993). Tissue Engineering. Science.

[3] Zong W., Shao X., Li J., Cai Z., Zhang X. (2024). Towards a biomimetic cellular structure and physical morphology with liposome-encapsulated agarose sol systems. Int. J. Biol. Macromol..

[4] Dhayal A., Kumar H., Mangla B., Singh D. (2025). Harnessing the power of carbohydrates: Chitosan and starch-based nanocomposites for sustainable developments. Inorg. Chem. Commun..

[5] Chen W., Dong T., Bai F., Wang J., Li X. (2022). Lignin–carbohydrate complexes, their fractionation, and application to healthcare materials: A review. Int. J. Biol. Macromol..

[6] Raposo C.D., Conceição C.A., Barros M.T. (2020). Nanoparticles Based on Novel Carbohydrate-Functionalized Polymers. Molecules.

[7] Gericke M., Witzler M., Enkelmann A., Schneider G., Schulze M., Heinze T. (2024). Functional Agarose Hydrogels Obtained by Employing Homogeneous Synthesis Strategies. Polysaccharides.

[8] Hu Y., Kim Y., Hong I., Kim M., Jung S. (2021). Fabrication of Flexible pH-Responsive Agarose/Succinoglycan Hydrogels for Controlled Drug Release. Polymers.

[9] Le K.V., Yamazaki K., Takemoto H., Ishiyama K., Naka Y., Sasaki T. (2023). Agarose hydrogels reinforced by chromonic aggregates. Mol. Cryst. Liq. Cryst..

[10] Liao J., Wang Y., Hou B., Zhang J., Huang H. (2023). Nano-chitin reinforced agarose hydrogels: Effects of nano-chitin addition and acidic gas-phase coagulation. Carbohydr. Polym..

[11] Wang X., Ao Y.-Y., Huang W., Liu B., An Y., Zhai M.-L. (2015). Radiation effects on physically cross-linked agarose hydrogels. Nucl. Sci. Tech..

[12] Arya N., Forget A., Shastri V.P. (2014). RGD-linked carboxylated agarose injectable hydrogels for cartilage tissue engineering. J. Tissue Eng. Regen. Med..

[13] Krömmelbein C., Xie X., Seifert J., Konieczny R., Friebe S., Käs J., Riedel S., Mayr S.G. (2022). Electron beam treated injectable agarose/alginate beads prepared by electrospraying. Carbohydr. Polym..

[14] Mokhtarzade A., Imani R., Shokrollahi P. (2023). A gradient four-layered gelatin methacrylate/agarose construct as an injectable scaffold for mimicking osteochondral tissue. J. Mater. Sci..

[15] Zhang Z., Wang X., Wang Y., Hao J. (2018). Rapid-Forming and Self-Healing Agarose-Based Hydrogels for Tissue Adhesives and Potential Wound Dressings. Biomacromolecules.

[16] Bao X., Hayashi K., Li Y., Teramoto A., Abe K. (2010). Novel agarose and agar fibers: Fabrication and characterization. Mater. Lett..

[17] Teng S.-H., Wang P., Kim H.-E. (2009). Blend fibers of chitosan–agarose by electrospinning. Mater. Lett..

[18] Bahcecioglu G., Hasirci N., Bilgen B., Hasirci V. (2019). Hydrogels of agarose, and methacrylated gelatin and hyaluronic acid are more supportive for in vitro meniscus regeneration than three dimensional printed polycaprolactone scaffolds. Int. J. Biol. Macromol..

[19] Ghaedamini S.L., Karbasi S., Hashemibeni B., Honarvar A., Rabiei A. (2023). PCL/Agarose 3D-printed scaffold for tissue engineering applications: Fabrication, characterization, and cellular activities. Res. Pharm. Sci..

[20] Marras-Marquez T., Peña J., Veiga-Ochoa M.D. (2024). Agarose as a tunable platform for oral drug delivery: Controlled release of a highly soluble drug, theophylline, within a sequentially-changing-pH medium. J. Drug Deliv. Sci. Technol..

[21] Ahmadi H., Pourmadadi M., Abdouss M., Rahdar A., Díez-Pascual A.M. (2023). Formulation of double nanoemulsions based on pH-sensitive poly acrylic acid/agarose/ZnO for quercetin controlled release. J. Mol. Liq..

[22] Witzler M., Ottensmeyer P.F., Gericke M., Heinze T., Tobiasch E., Schulze M. (2019). Non-Cytotoxic Agarose/Hydroxyapatite Composite Scaffolds for Drug Release. Int. J. Mol. Sci..

[23] Singh Y.P., Bhardwaj N., Mandal B.B. (2016). Potential of Agarose/Silk Fibroin Blended Hydrogel for in Vitro Cartilage Tissue Engineering. ACS Appl. Mater. Interfaces.

[24] Watanabe J., Kashii M., Hirao M., Oka K., Sugamoto K., Yoshikawa H., Akashi M. (2007). Quick-forming hydroxyapatite/agarose gel composites induce bone regeneration. J. Biomed. Mater. Res. Part A.

[25] Kazimierczak P., Palka K., Przekora A. (2019). Development and Optimization of the Novel Fabrication Method of Highly Macroporous Chitosan/Agarose/Nanohydroxyapatite Bone Scaffold for Potential Regenerative Medicine Applications. Biomolecules.

[26] Gomer R.H., Pilling D., Kauvar L.M., Ellsworth S., Ronkainen S.D., Roife D., Davis S.C. (2009). A serum amyloid P-binding hydrogel speeds healing of partial thickness wounds in pigs. Wound Repair Regen..

[27] Uno N., Li Z., Liu C. (2023). Single-tube one-step gel-based RT-RPA/PCR for highly sensitive molecular detection of HIV. Analyst.

[28] Qian C., Xiao Y., Wang J., Li Y., Li S., Wei B., Du W., Feng X., Chen P., Liu B.-F. (2021). Rapid exosomes concentration and in situ detection of exosomal microRNA on agarose-based microfluidic chip. Sens. Actuators B Chem..

[29] Wang M., Mahajan A., Miller J.S., McKenna D.H., Aksan A. (2023). Physicochemical Mechanisms of Protection Offered by Agarose Encapsulation during Cryopreservation of Mammalian Cells in the Absence of Membrane-Penetrating Cryoprotectants. ACS Appl. Bio Mater..

[30] Su C.-R., Yu S.-S., Zhao J.-M., Yang J., Dong L.-Y., Wang X.-H. (2024). Fabrication of micron-sized boronate-decorated polyethyleneimine-grafted magnetic agarose beads for specific enrichment of ribonucleic acid. J. Chromatogr. A.

[31] Zarrintaj P., Manouchehri S., Ahmadi Z., Saeb M.R., Urbanska A.M., Kaplan D.L., Mozafari M. (2018). Agarose-based biomaterials for tissue engineering. Carbohydr. Polym..

[32] Jiang C., Liu Z., Cheng D., Mao X. (2020). Agarose degradation for utilization: Enzymes, pathways, metabolic engineering methods and products. Biotechnol. Adv..

[33] Najihah A.Z., Hassan M.Z., Ismail Z. (2024). Current trend on preparation, characterization and biomedical applications of natural polysaccharide-based nanomaterial reinforcement hydrogels: A review. Int. J. Biol. Macromol..

[34] Huang Y., Peng S., Chen Y., Chu B. (2025). Agarose Hydrogels for Bone Tissue Engineering, from Injectables to Bioprinting. Gels.

[35] Chung J.T., Lau C.M.L., Chau Y. (2021). The effect of polysaccharide-based hydrogels on the response of antigen-presenting cell lines to immunomodulators. Biomater. Sci..

[36] Wei W., Zhang Q., Zhou W., Liu Z., Wang Y., Alakpa E.V., Ouyang H., Liu H. (2019). Immunomodulatory application of engineered hydrogels in regenerative medicine. Appl. Mater. Today.

[37] Omidian H., Chowdhury S.D., Akhzarmehr A. (2024). Hydrogels in biosensing and medical diagnostics. J. Bioact. Compat. Polym..

[38] Wang Y., Li R., Shu W., Chen X., Lin Y., Wan J. (2024). Designed Nanomaterials-Assisted Proteomics and Metabolomics Analysis for In Vitro Diagnosis. Small Methods.

[39] Senior J.J., Moakes R.J.A., Cooke M.E., Moxon S.R., Smith A.M., Grover L.M. (2023). Agarose Fluid Gels Formed by Shear Processing During Gelation for Suspended 3D Bioprinting. J. Vis. Exp..

[40] Liu X., Hu Y., Ju Y., Yang P., Shen N., Yang A., Wu R., Fang B., Liu L. (2024). Immunomodulatory hydrogels for tissue repair and regeneration. APL Mater..

[41] Venkatachalam G., Giri J., Mallik S., Arumugam G.S., Arulmani M., Dewangan V.K., Doble M., Zhao Z. (2024). Immunomodulatory zymosan/ι-carrageenan/agarose hydrogel for targeting M2 to M1 macrophages (antitumoral). RSC Adv..

[42] Tan Y., Richards D.J., Trusk T.C., Visconti R.P., Yost M.J., Kindy M.S., Drake C.J., Argraves W.S., Markwald R.R., Mei Y. (2014). 3D printing facilitated scaffold-free tissue unit fabrication. Biofabrication.

[43] Budharaju H., Chandrababu H., Zennifer A., Chellappan D., Sethuraman S., Sundaramurthi D. (2024). Tuning thermoresponsive properties of carboxymethyl cellulose (CMC)–agarose composite bioinks to fabricate complex 3D constructs for regenerative medicine. Int. J. Biol. Macromol..

[44] Sreepadmanabh M., Ganesh M., Bhat R., Bhattacharjee T. (2023). Jammed microgel growth medium prepared by flash-solidification of agarose for 3D cell culture and 3D bioprinting. Biomed. Mater..

[45] Peitso V., Sarmadian Z., Henriques J., Lauwers E., Paggi C.A., Mobasheri A. (2024). Development of a Microphysiological Cartilage-on-Chip Platform for Dynamic Biomechanical Stimulation of Three-Dimensional Encapsulated Chondrocytes in Agarose Hydrogels. Curr. Protoc..

[46] Wang D., Li Q., Zhou C., Li Z., Lu K., Liu Y., Xuan L., Wang X. (2024). Dissolvable temporary barrier: A novel paradigm for flexible hydrogel patterning in organ-on-a-chip models. Bio-Des. Manuf..

[47] Tan J., Jia S., Xu Q., Lin C., Cao Y., Shen J., Han S., Li Z., Zhou X. (2024). Hydrogel encapsulation facilitates a low-concentration cryoprotectant for cryopreservation of mouse testicular tissue. Colloids Surf. B Biointerfaces.

[48] Tang Z., Wang Y., Yang Z., Chen J., Cai S., Zhang Z., Wang J., Wei Z. (2025). Hydrogel microcapsulation technology reduces the DMSO concentration required for stem cell cryopreservation. Regen. Ther..

[49] Zhang C., Zhou Y., Zhang L., Wu L., Chen Y., Xie D., Chen W. (2018). Hydrogel Cryopreservation System: An Effective Method for Cell Storage. Int. J. Mol. Sci..

[50] Ruta A., Krishnan K., Elisseeff J.H. (2024). Single-cell transcriptomics in tissue engineering and regenerative medicine. Nat. Rev. Bioeng..

[51] Letherby M.R., Young D.A. (1981). The gelation of agarose. J. Chem. Soc. Faraday Trans. 1.

[52] Xiong J.-Y., Narayanan J., Liu X.-Y., Chong T.K., Chen S.B., Chung T.-S. (2005). Topology Evolution and Gelation Mechanism of Agarose Gel. J. Phys. Chem. B.

[53] Kildeeva N.R., Privar Y.O., Bratskaya S.Y. (2025). Crosslinking Agents in the Targeted Design of Chitosan-Based Materials. Colloid J..

[54] Horinaka J.-I., Ogawa S. (2023). Cyclic deformation behavior of agarose hydrogels prepared at different gelation concentrations. Int. J. Biol. Macromol..

[55] Ed-Daoui A., Snabre P. (2021). Poroviscoelasticity and compression-softening of agarose hydrogels. Rheol. Acta.

[56] Li C., Arakawa T. (2019). Agarose native gel electrophoresis of proteins. Int. J. Biol. Macromol..

[57] Zhang Y., Deng Z., Lou D., Wang Y., Wang R., Hu R., Zhang X., Zhu Q., Chen Y., Liu F. (2020). High-Efficiency Separation of Extracellular Vesicles from Lipoproteins in Plasma by Agarose Gel Electrophoresis. Anal. Chem..

[58] Sakuma C., Sato T., Shibata T., Nakagawa M., Kurosawa Y., Okumura C.J., Maruyama T., Arakawa T., Akuta T. (2021). Western blotting analysis of proteins separated by agarose native gel electrophoresis. Int. J. Biol. Macromol..

[59] Creutzburg S.C.A., Swartjes T., van der Oost J. (2020). Medium-throughput in vitro detection of DNA cleavage by CRISPR-Cas12a. Methods.

[60] Chaga G., Bochkariov D.E., Jokhadze G.G., Hopp J., Nelson P. (1999). Natural poly-histidine affinity tag for purification of recombinant proteins on cobalt(II)-carboxymethylaspartate crosslinked agarose. J. Chromatogr. A.

[61] Sedykh S.E., Purvinsh L.V., Burkova E.E., Dmitrenok P.S., Ryabchikova E.I., Nevinsky G.A. (2022). Analysis of Proteins and Peptides of Highly Purified CD9+ and CD63+ Horse Milk Exosomes Isolated by Affinity Chromatography. Int. J. Mol. Sci..

[62] Konnova S., Fakhrullin R. (2017). Fabrication of Magnetically Responsive Agarose Microbeads Doped with Live Microbial Cells. BioNanoScience.

[63] Eun Y.-J., Utada A.S., Copeland M.F., Takeuchi S., Weibel D.B. (2011). Encapsulating Bacteria in Agarose Microparticles Using Microfluidics for High-Throughput Cell Analysis and Isolation. ACS Chem. Biol..

[64] Peng J., Cao J., Wang L., Guo Z., Hou X. (2024). A portable hydrogel kit based on Au@GM88A/I combined with mobile phone for polychromatic semi-quantitative and quantitative sensing analysis. Biosens. Bioelectron..

[65] Kim D.H., Hur J., Park H.G., Kim M.I. (2017). Reagentless colorimetric biosensing platform based on nanoceria within an agarose gel matrix. Biosens. Bioelectron..

[66] De Beuckeleer S., Vanhooydonck A., Van Den Daele J., Van De Looverbosch T., Asselbergh B., Kim H., Campsteijn C., Ponsaerts P., Watts R., De Vos W.H. (2025). An agarose fluidic chip for high-throughput in toto organoid imaging. Lab Chip.

[67] Xiao Q., Chen G., Zhang Y.-H., Chen F.-Q., Weng H.-F., Xiao A.-F. (2021). Agarose Stearate-Carbomer940 as Stabilizer and Rheology Modifier for Surfactant-Free Cosmetic Formulations. Mar. Drugs.

[68] Zanotti A., Baldino L., Cardea S., Reverchon E. (2024). Production of Agarose-Hydroxyapatite Composites via Supercritical Gel Drying, for Bone Tissue Engineering. Molecules.

[69] Batumalay M., Harun S.W., Ahmad F., Nor R.M., Zulkepely N.R., Ahmad H. (2014). Study of a fiber optic humidity sensor based on agarose gel. J. Mod. Opt..

[70] Gavira J.A., García-Ruiz J.M. (2002). Agarose as crystallisation media for proteins II: Trapping of gel fibres into the crystals. Acta Crystallogr. D Biol. Crystallogr..

[71] Guo X., Chen Y., Ji W., Chen X., Li C., Ge R. (2019). Enrichment of cancer stem cells by agarose multi-well dishes and 3D spheroid culture. Cell Tissue Res..

[72] Sedykh S.E., Purvinish L.V., Burkova E.E., Dmitrenok P.S., Vlassov V.V., Ryabchikova E.I., Nevinsky G.A. (2021). Analysis of peptides and small proteins in preparations of horse milk exosomes, purified on anti-CD81-Sepharose. Int. Dairy J..

[73] Tu Y., Yuan J., Lei D., Tan H., Wei J., Huang W., Zhang L. (2018). TiO_2_-pattern-modulated actuation of an agarose@CNT/agarose bilayer induced by light and humidity. J. Mater. Chem. A.

[74] Shu Q., Gu Y., Xia W., Lu X., Pang Y., Teng J., Liu B., Li Y. (2024). Injectable hydrogels for bioelectronics: A viable alternative to traditional hydrogels. Chem. Eng. J..

[75] Wang S., Yan X., Jiao X., Yang H. (2024). Experimental Study of the Implantation Process for Array Electrodes into Highly Transparent Agarose Gel. Materials.

[76] Yang H. (2013). Chemically Modified Sepharose as Support for the Immobilization of Cholesterol Oxidase. J. Microbiol. Biotechnol..

[77] Xu H., Wei B., Liu X., Huang Y., Zhou W., Liang H. (2022). Robust enhancing stability and fructose tolerance of sucrose phosphorylase by immobilization on Ni-NTA functionalized agarose microspheres for the biosynthesis of 2-α-glucosylglycerol. Biochem. Eng. J..

[78] Kantak M., Shende P. (2022). Nucleic Acid-conjugated Carbohydrate Nanobiosensors: A Multimodal Tool for Disease Diagnosis. Curr. Pharm. Des..

[79] Vashist S.K. (2021). Trends in Multiplex Immunoassays for In Vitro Diagnostics and Point-of-Care Testing. Diagnostics.

[80] Zhang Y., Cheng Y., Zhao C., Chen J., Wu Z., Wang J., Wang D. (2025). Simultaneous detection of protein and nucleic acid biomarkers with a CRISPR-based assay. Chem. Eng. J..

[81] Ren X., Sun Y., Li S., Bai J., Gao X., Zou G. (2025). Direct and Glow Chemiluminescence of Dual-Stabilizer-Capped Au Nanocrystals for Automatic Immunoassays. Anal. Chem..

[82] Li J., Mo L., Lu C.-H., Fu T., Yang H.-H., Tan W. (2016). Functional nucleic acid-based hydrogels for bioanalytical and biomedical applications. Chem. Soc. Rev..

[83] Cano-Gamez K., Maclean P., Inoue M., Hussainy S., Foss E., Wainwright C., Qin H., McKechnie S., Song C.-X., Knight J.C. (2025). The circulating cell-free DNA landscape in sepsis is dominated by impaired liver clearance. Cell Genom..

[84] Dutta S., Dutta S., Somanath P.R., Narayanan S.P., Wang X., Zhang D. (2025). Circulating Nucleosomes and Histones in the Development of Lung Injury and Sepsis. Curr. Issues Mol. Biol..

[85] Theilen H., Koch T., Ragaller M. (2007). Mikrozirkulation bei Sepsis und septischem Schock. Hamostaseologie.

[86] Feng Q., Ai Y.-H., Gong H., Wu L., Ai M.-L., Deng S.-Y., Huang L., Peng Q.-Y., Zhang L.-N. (2019). Characterization of Sepsis and Sepsis-Associated Encephalopathy. J. Intensive Care Med..

[87] Rudd K.E., Johnson S.C., Agesa K.M., Shackelford K.A., Tsoi D., Kievlan D.R., Colombara D.V., Ikuta K.S., Kissoon N., Finfer S. (2020). Global, regional, and national sepsis incidence and mortality, 1990–2017: Analysis for the Global Burden of Disease Study. Lancet.

[88] García-Giménez J.L., García-López E., Mena-Mollá S., Beltrán-García J., Osca-Verdegal R., Nacher-Sendra E., Aguado-Velasco C., Casabó-Vallés G., Romá-Mateo C., Rodriguez-Gimillo M. (2023). Validation of circulating histone detection by mass spectrometry for early diagnosis, prognosis, and management of critically ill septic patients. J. Transl. Med..

[89] Shahriari S., Selvaganapathy P.R. (2024). A Fully Integrated Microfluidic Device with Immobilized Dyes for Simultaneous Detection of Cell-Free DNA and Histones from Plasma Using Dehydrated Agarose Gates. Gels.

[90] Mehri M., Mehrizi A.A., Ajoudanian M., Fathi S., Mohammadi S., Sharifi K. (2025). A novel approach to rapid fabrication of robust paper-based microfluidic devices using FDM 3D printing. Microsyst. Technol..

[91] Xie M., Fu Z., Lu C., Wu S., Pan L., He Y., Sun Y., Wang J. (2024). Rapid fabrication of modular 3D paper-based microfluidic chips using projection-based 3D printing. Bio-Des. Manuf..

[92] Marras-Marquez T., Peña J., Veiga-Ochoa M.D. (2014). Agarose drug delivery systems upgraded by surfactants inclusion: Critical role of the pore architecture. Carbohydr. Polym..

[93] Lim G., Kim H.R., Yoo C., Oh Y.K., Park Y.S., Park S., Jang S., Priscilla L., Lee D.Y. (2025). Mucoadhesive and Self-Assembled Chitosan Oligosaccharide-Glycyrrhizin Nanoparticle for Oral Nanotherapeutic in Inflammatory Bowel Disease. ACS Nano.

[94] Di J., Li J., Sun C., Xu L., Li X. (2025). Advances in Cellulose-Based Hydrogels for Drug Delivery: Preparation, Modification and Challenges. Gels.

[95] Vlad R.-A., Pintea A., Pintea C., Rédai E.-M., Antonoaea P., Bîrsan M., Ciurba A. (2025). Hydroxypropyl Methylcellulose—A Key Excipient in Pharmaceutical Drug Delivery Systems. Pharmaceutics.

[96] Raj A.A., Sundaramoorthy S. (2025). Doxorubicin-loaded pH-responsive amine functionalized cellulose nanogel to treat cervical cancer through topical drug delivery. J. Bioact. Compat. Polym..

[97] Goyal P., Malviya R., Awasthi R., Warsi M.H. (2025). Jute fiber crystalline cellulose grafted polysaccharide: Preparation, grafting and optimization for drug delivery applications. Int. J. Biol. Macromol..

[98] Aguiar A.C., Bianchi J.R.O., Lopes J.H., Ferreira F.V., Lona L.M.F. (2025). Nanocellulose-Based Capsules with pH Responsiveness for Colon-Targeted Curcumin Delivery. ACS Appl. Nano Mater..

[99] Tang L., Hong B., Li T., Huang B. (2019). Development of bilayer films based on shellac and esterified cellulose nanocrystals for buccal drug delivery. Cellulose.

[100] Jalalibidgoli F., Irankhahi P., Hajihassani H., Ghotbi-Ravandi A.A. (2025). Innovative Applications of Marine Macroalgae Polysaccharides in Tissue Engineering and Drug Delivery: A Review Study. Health Sci. Rep..

[101] Mottaghitalab F., Khodadadi Yazdi M., Saeb M.R., Bączek T., Farokhi M. (2024). Green and sustainable hydrogels based on quaternized chitosan to enhance wound healing. Chem. Eng. J..

[102] Gong J., Hou L., Ching Y.C., Ching K.Y., Hai N.D., Chuah C.H. (2024). A review of recent advances of cellulose-based intelligent-responsive hydrogels as vehicles for controllable drug delivery system. Int. J. Biol. Macromol..

[103] Neumann M., di Marco G., Iudin D., Viola M., van Nostrum C.F., van Ravensteijn B.G.P., Vermonden T. (2023). Stimuli-Responsive Hydrogels: The Dynamic Smart Biomaterials of Tomorrow. Macromolecules.

[104] Juthi A.Z., Li F., Wang B., Alam M.M., Talukder M.E., Qiu B. (2023). pH-Responsive Super-Porous Hybrid Hydrogels for Gastroretentive Controlled-Release Drug Delivery. Pharmaceutics.

[105] Luo Y., Yin X., Yin X., Chen A., Zhao L., Zhang G., Liao W., Huang X., Li J., Zhang C.Y. (2019). Dual pH/Redox-Responsive Mixed Polymeric Micelles for Anticancer Drug Delivery and Controlled Release. Pharmaceutics.

[106] Pan Y., Ma X., Wu Y., Zhao Z., He Q., Ji Y. (2024). pH-responsive glucose-powered Janus polymer brushes nanomotors for drug delivery and controlled release. Colloids Surf. A Physicochem. Eng. Asp..

[107] Xiang S., Guilbaud-Chéreau C., Hoschtettler P., Stefan L., Bianco A., Ménard-Moyon C. (2024). Preparation and optimization of agarose or polyacrylamide/amino acid-based double network hydrogels for photocontrolled drug release. Int. J. Biol. Macromol..

[108] Kolanthai E., Colon V.S.D., Sindu P.A., Chandra V.S., Karthikeyan K.R., Babu M.S., Sundaram S.M., Palanichamy M., Kalkura S.N. (2015). Effect of solvent; enhancing the wettability and engineering the porous structure of a calcium phosphate/agarose composite for drug delivery. RSC Adv..

[109] Guo Y., Wang Y., Chen H., Jiang W., Zhu C., Toufouki S., Yao S. (2022). A new deep eutectic solvent-agarose gel with hydroxylated fullerene as electrical “switch” system for drug release. Carbohydr. Polym..

[110] Dutcher M., Chewchuk S., Benavente-Babace A., Soucy N., Wan F., Merrett K., Davis D.R., Harden J.L., Godin M. (2023). Encapsulating therapeutic cells in RGD-modified agarose microcapsules. Biomed. Mater..

[111] Forget A., Shastri V.P. (2025). Sulfated and Phosphorylated Agarose as Biomaterials for a Biomimetic Paradigm for FGF-2 Release. Biomimetics.

[112] Philip A.K., Samuel B.A., Saleh Y.S., Bhatia S., Mohammad B.I., Al-Aubaidy H.A. (2025). pH-responsive agarose hydrogel for enhanced gastrointestinal ibuprofen delivery: A novel pressure-sensitive adhesive system. Next Mater..

[113] Kolanthai E., Sindu P.A., Arul K.T., Chandra V.S., Manikandan E., Kalkura S.N. (2017). Agarose encapsulated mesoporous carbonated hydroxyapatite nanocomposites powder for drug delivery. J. Photochem. Photobiol. B Biol..

[114] Pourmadadi M., Omrani Z., Doustgani A., Yazdian F., Nourhashemi M., Ahmadi T., Rahdar A., Fathi-karkan S., Valizadeh N., Ferreira L.F.R. (2025). Machine learning models predict pH-responsive drug release from liver-targeted agarose-CMC-CeO_2_ nanocomposites: Liver-targeted antioxidant strike. J. Drug Deliv. Sci. Technol..

[115] Kim C., Jeong D., Kim S., Kim Y., Jung S. (2019). Cyclodextrin functionalized agarose gel with low gelling temperature for controlled drug delivery systems. Carbohydr. Polym..

[116] Mohamadzadeh F., Pourmadadi M., Mazinani S., Abdouss M., Rahdar A., Pandey S. (2026). Effect of Double Microemulsion on the Aga/CMC/MoS_2_ Nano Composites for Drug Delivery: Green Synthesis and Physico-Chemical Characterization. Nano Life.

[117] Syed M.A., Hanif S., Ain N.U., Syed H.K., Zahoor A.F., Khan I.U., Abualsunun W.A., Jali A.M., Qahl S.H., Sultan M.H. (2022). Assessment of Binary Agarose–Carbopol Buccal Gels for Mucoadhesive Drug Delivery: Ex Vivo and In Vivo Characterization. Molecules.

[118] Ning L., You C., Zhang Y., Li X., Wang F. (2021). Polydopamine loaded fluorescent nanocellulose–agarose hydrogel: A pH-responsive drug delivery carrier for cancer therapy. Compos. Commun..

[119] Aslam M., Barkat K., Malik N.S., Alqahtani M.S., Anjum I., Khalid I., Tulain U.R., Gohar N., Zafar H., Paiva-Santos A.C. (2022). pH Sensitive Pluronic Acid/Agarose-Hydrogels as Controlled Drug Delivery Carriers: Design, Characterization and Toxicity Evaluation. Pharmaceutics.

[120] Rajabzadeh-Khosroshahi M., Pourmadadi M., Yazdian F., Rashedi H., Navaei-Nigjeh M., Rasekh B. (2022). Chitosan/agarose/graphitic carbon nitride nanocomposite as an efficient pH-sensitive drug delivery system for anticancer curcumin releasing. J. Drug Deliv. Sci. Technol..

[121] Bayat F., Pourmadadi M., Eshaghi M.M., Yazdian F., Rashedi H. (2023). Improving Release Profile and Anticancer Activity of 5-Fluorouracil for Breast Cancer Therapy Using a Double Drug Delivery System: Chitosan/Agarose/γ-Alumina Nanocomposite@Double Emulsion. J. Clust. Sci..

[122] Pinelli F., Ponti M., Delleani S., Pizzetti F., Vanoli V., Vangosa F.B., Castiglione F., Haugen H., Nogueira L.P., Rossetti A. (2023). β-Cyclodextrin functionalized agarose-based hydrogels for multiple controlled drug delivery of ibuprofen. Int. J. Biol. Macromol..

[123] Rajaei M., Rashedi H., Yazdian F., Navaei-Nigjeh M., Rahdar A., Díez-Pascual A.M. (2023). Chitosan/agarose/graphene oxide nanohydrogel as drug delivery system of 5-fluorouracil in breast cancer therapy. J. Drug Deliv. Sci. Technol..

[124] Abdollahi H., Amiri S., Amiri F., Moradi S., Zarrintaj P. (2024). Antibacterial Biocomposite Based on Chitosan/Pluronic/Agarose Noncovalent Hydrogel: Controlled Drug Delivery by Alginate/Tetracycline Beads System. J. Funct. Biomater..

[125] Najafi M., Khoddam Z., Masnavi M., Pourmadadi M., Abdouss M. (2024). Physicochemical and in vitro characterization of Agarose based nanocarriers incorporated with Graphene Quantum Dots/α-Fe_2_O_3_ for targeted drug delivery of Quercetin to liver cancer treatment. Mater. Chem. Phys..

[126] Zhang Y., Liang L., Kou Q., Sun H., He Y., Li L., Shi C. (2025). Multimodal Imaging Nanoassembled Agarose Microspheres for Drug Delivery in Transarterial Chemoembolization. ACS Appl. Bio Mater..

[127] Falahatpisheh S., Naghib S.M., Naimi-Jamal M.R., Jafari K.M., Sartipzadeh O. (2025). Chitosan/agarose-encapsulated oleic acid-coated magnetite nanoparticles as a chemotherapeutic-loaded scaffold for drug delivery: Physico-chemical and in vitro biological characteristics. Int. J. Biol. Macromol..

[128] Zhang J., Yang F., Wu H., Ong H.L., Arnold P., Zhang M., Jiang Y., Bahar D., Yuan Z., Yang X. (2025). Wearable transdermal drug delivery system controlled by wirelessly powered acoustic waves. J. Control. Release.

[129] Irastorza-Lorenzo A., Sánchez-Porras D., Ortiz-Arrabal O., de Frutos M.J., Esteban E., Fernández J., Janer A., Campos A., Campos F., Alaminos M. (2021). Evaluation of Marine Agarose Biomaterials for Tissue Engineering Applications. Int. J. Mol. Sci..

[130] Zhan Y.-L., Wen K.-C., Li Z.-A., Zang J., Sun P., Li F.-Q. (2024). Microwave assisted black phosphorus-based core-shell composites with synergistic antibacterial and osteogenic ability for bone tissue repair. Compos. Part B Eng..

[131] Yang B., Liang C., Chen D., Cheng F., Zhang Y., Wang S., Shu J., Huang X., Wang J., Xia K. (2022). A conductive supramolecular hydrogel creates ideal endogenous niches to promote spinal cord injury repair. Bioact. Mater..

[132] Guo Y., Tian B., Xie Y., Xiao J. (2025). Physically crosslinked agarose–hyaluronic acid hydrogel for injectable treatment of photoaged skin. J. Mater. Chem. B.

[133] Peng Y., Lu M., Zhou Z., Wang C., Liu E., Zhang Y., Liu T., Zuo J. (2022). Natural biopolymer scaffold for meniscus tissue engineering. Front. Bioeng. Biotechnol..

[134] Chu B., Zhang A., Huang J., Peng X., You L., Wu C., Tang S. (2020). Preparation and biological evaluation of a novel agarose-grafting-hyaluronan scaffold for accelerated wound regeneration. Biomed. Mater..

[135] Hasan M.L., Padalhin A.R., Kim B., Lee B.-T. (2019). Preparation and evaluation of BCP-CSD-agarose composite microsphere for bone tissue engineering. J. Biomed. Mater. Res. Part B Appl. Biomater..

[136] Lin H., Cheng A.W.-M., Alexander P.G., Beck A.M., Tuan R.S. (2014). Cartilage Tissue Engineering Application of Injectable Gelatin Hydrogel with In Situ Visible-Light-Activated Gelation Capability in Both Air and Aqueous Solution. Tissue Eng. Part A.

[137] Ng K.W., Kugler L.E., Doty S.B., Ateshian G.A., Hung C.T. (2009). Scaffold degradation elevates the collagen content and dynamic compressive modulus in engineered articular cartilage. Osteoarthr. Cartil..

[138] Sartip E., Behzad T., Kharaziha M. (2025). Fabrication and characterization of 3D printed agarose/poly(ethylene glycol) diacrylate/hydroxyapatite nanocomposite hydrogel for cartilage tissue engineering. Int. J. Biol. Macromol..

[139] Bavaro T., Tengattini S., Rezwan R., Chiesa E., Temporini C., Dorati R., Massolini G., Conti B., Ubiali D., Terreni M. (2021). Design of epidermal growth factor immobilization on 3D biocompatible scaffolds to promote tissue repair and regeneration. Sci. Rep..

[140] Stefani R.M., Lee A.J., Tan A.R., Halder S.S., Hu Y., Guo X.E., Stoker A.M., Ateshian G.A., Marra K.G., Cook J.L. (2020). Sustained low-dose dexamethasone delivery via a PLGA microsphere-embedded agarose implant for enhanced osteochondral repair. Acta Biomater..

[141] Hu J.-X., Ran J.-B., Chen S., Jiang P., Shen X.-Y., Tong H. (2016). Carboxylated Agarose (CA)-Silk Fibroin (SF) Dual Confluent Matrices Containing Oriented Hydroxyapatite (HA) Crystals: Biomimetic Organic/Inorganic Composites for Tibia Repair. Biomacromolecules.

[142] Zhang H., He X., Zhang Y., Li Q., Liu Y., Zhang Y., Wang Z., Zhu Q., Li X. (2021). Shapable bulk agarose–gelatine–hydroxyapatite–minocycline nanocomposite fabricated using a mineralizing system aided with electrophoresis for bone tissue regeneration. Biomed. Mater..

[143] Xu Y., Huang Y., Armstrong J., Xu W., Biglino G., Zhang W., Qi Q., Chen X., Abram S., Vyas C. (2026). Recent advances in biomaterials and structural design of 3D printed multiphasic scaffolds for osteochondral regeneration. Mater. Sci. Eng. R Rep..

[144] Shepherd D.E.T., Seedhom B.B. (1997). A technique for measuring the compressive modulus of articular cartilage under physiological loading rates with preliminary results. Proc. Inst. Mech. Eng. Part H.

[145] Amhare A.F., Qiao L., Deng H., Lin J., Wang J., Wang W., Han J. (2025). The current status of nano-hydrogel preparations for osteochondral repair: Systematic Review. Front. Bioeng. Biotechnol..

[146] Bu W., Wu Y., Ghaemmaghami A.M., Sun H., Mata A. (2022). Rational design of hydrogels for immunomodulation. Regen. Biomater..

[147] Cai H., Chen A., You Y., Qu J., Scheper V., Tang J., Zhang H. (2025). Implantable drug delivery systems with a bioinspired zwitterionic nanocoating resist foreign body reaction-induced obstruction and enable sustained delivery. Biomater. Sci..

[148] Tian J., Peng J., Hu C., Lei S., Wu D. (2024). A general strategy for prepared multifunction double-ions agarose hydrogel dressing promotes wound healing. Mater. Des..

[149] Su T., Zhang M., Zeng Q., Pan W., Huang Y., Qian Y., Dong W., Qi X., Shen J. (2021). Mussel-inspired agarose hydrogel scaffolds for skin tissue engineering. Bioact. Mater..

[150] Ni D., Zhou H., Wang P., Xu F., Li C. (2023). Visualizing Macrophage Phenotypes and Polarization in Diseases: From Biomarkers to Molecular Probes. Phenomics.

[151] Kadlec M., Pekař M., Smilek J. (2024). Mechanical properties of agarose hydrogels tuned by amphiphilic structures. Colloids Surf. A Physicochem. Eng. Asp..

[152] Yin Y., He X.-T., Wang J., Wu R.-X., Xu X.-Y., Hong Y.-L., Tian B.-M., Chen F.-M. (2020). Pore size-mediated macrophage M1-to-M2 transition influences new vessel formation within the compartment of a scaffold. Appl. Mater. Today.

[153] Afrose N., Chakraborty R., Rajendran K. (2025). Plasma-polymerized chitosan/agarose/chitosan-agarose composite coatings for antibacterial and antibiofilm wound dressings: A review. Int. J. Biol. Macromol..

[154] Xu L.Q., Li N.N., Chen J.C., Fu G.D., Kang E.-T. (2015). Quaternized poly(2-(dimethylamino)ethyl methacrylate)-grafted agarose copolymers for multipurpose antibacterial applications. RSC Adv..

[155] Dsouza A., Taylor D., Parmenter C., Hand R.A., Brettschneider J., Unnikrishnan M., Constantinidou C., Charmet J. (2026). Dynamic bacterial growth modulation in structurally distinct and functionally tuneable agarose hydrogels. Commun. Mater..

[156] Liu B., Luo H. (2023). Effects of Ag Nanoparticles Impregnated over Chitosan-Agarose Modified Magnetic Nanocomposite as an Efficient Reusable Nano Catalyst on Bone Regeneration in a Rat Calvarial Defect Model and Screening System. J. Biomed. Nanotechnol..

[157] Whaley D., Damyar K., Witek R.P., Mendoza A., Alexander M., Lakey J.R.T. (2021). Cryopreservation: An Overview of Principles and Cell-Specific Considerations. Cell Transplant..

[158] Ntai A., La Spada A., De Blasio P., Biunno I. (2018). Trehalose to cryopreserve human pluripotent stem cells. Stem Cell Res..

[159] Svalgaard J.D., Munthe-Fog L., Rivera Ballesteros O., Brooks P.T., Rangatchew F., Vester-Glowinski P.V., Haastrup E.K., Fischer-Nielsen A. (2020). Cryopreservation of adipose-derived stromal/stem cells using 1–2% Me_2_SO (DMSO) in combination with pentaisomaltose: An effective and less toxic alternative to comparable freezing media. Cryobiology.

[160] Haastrup E.K., Munthe-Fog L., Rivera Ballesteros O., Fischer-Nielsen A., Svalgaard J.D. (2021). DMSO (Me_2_SO) concentrations of 1–2% in combination with pentaisomaltose are effective for cryopreservation of T cells. Transfus. Apher. Sci..

[161] Roesch A., Windisch R., Wichmann C., Wolkers W.F., Kersten G., Menzen T. (2025). Reducing dimethyl sulfoxide content in Jurkat cell formulations suitable for cryopreservation. Cryobiology.

[162] Murray A., Kilbride P., Gibson M.I. (2024). Trehalose in cryopreservation. Applications, mechanisms and intracellular delivery opportunities. RSC Med. Chem..

[163] Mazurek Ł., Kuś M., Jurak J., Rybka M., Kuczeriszka M., Stradczuk-Mazurek M., Konop M. (2025). Biomedical potential of alginate wound dressings—From preclinical studies to clinical applications: A review. Int. J. Biol. Macromol..

[164] U.S. Food and Drug Administration 510(k) Premarket Notification K904488: KALTOSTAT Wound Dressing. https://www.accessdata.fda.gov/scripts/cdrh/cfdocs/cfpmn/pmn.cfm?ID=K904488.

[165] Clegg J.R., Adebowale K., Zhao Z., Mitragotri S. (2024). Hydrogels in the clinic: An update. Bioeng. Transl. Med..

[166] Correa S., Grosskopf A.K., Lopez Hernandez H., Chan D., Yu A.C., Stapleton L.M., Appel E.A. (2021). Translational Applications of Hydrogels. Chem. Rev..

[167] Zöller K., To D., Bernkop-Schnürch A. (2025). Biomedical applications of functional hydrogels: Innovative developments, relevant clinical trials and advanced products. Biomaterials.

[168] Huang T., Sun Z., Heath D.E., O’Brien-Simpson N., O’Connor A.J. (2024). 3D printed and smart alginate wound dressings with pH-responsive drug and nanoparticle release. Chem. Eng. J..

[169] Johari N., Rahimi F., Azami H., Rafati F., Nokhbedehghan Z., Samadikuchaksaraei A., Moroni L. (2024). The impact of copper nanoparticles surfactant on the structural and biological properties of chitosan/sodium alginate wound dressings. Biomater. Adv..

[170] Zohari F., Johari N., Zare M., Nokhbedehghan Z., Samadikuchaksaraei A. (2025). Integrating magnesium MOF and curcumin into chitosan/sodium alginate wound dressings: Insights into structural, mechanical, and biological impacts. Cellulose.

[171] Latiyan S., Kumar T.S.S., Doble M., Kennedy J.F. (2023). Perspectives of nanofibrous wound dressings based on glucans and galactans—A review. Int. J. Biol. Macromol..

[172] Sawant S., Salunke B., Taylor L., Kim B. (2017). Enhanced Agarose and Xylan Degradation for Production of Polyhydroxyalkanoates by Co-Culture of Marine Bacterium, Saccharophagus degradans and Its Contaminant, Bacillus cereus. Appl. Sci..

[173] Place E.S., Evans N.D., Stevens M.M. (2009). Complexity in biomaterials for tissue engineering. Nat. Mater..

[174] Hoffman A.S. (2012). Hydrogels for biomedical applications. Adv. Drug Deliv. Rev..

[175] Li J., Mooney D.J. (2016). Designing hydrogels for controlled drug delivery. Nat. Rev. Mater..

[176] Li X., Gong J.P. (2024). Design principles for strong and tough hydrogels. Nat. Rev. Mater..

